# Activin-A impedes the establishment of CD4^+^ T cell exhaustion and enhances anti-tumor immunity in the lung

**DOI:** 10.1186/s13046-021-02092-5

**Published:** 2021-09-21

**Authors:** Ioannis Morianos, Aikaterini Tsitsopoulou, Konstantinos Potaris, Dimitrios Valakos, Ourania Fari, Giannis Vatsellas, Clementine Bostantzoglou, Andreas Photiades, Mina Gaga, Georgina Xanthou, Maria Semitekolou

**Affiliations:** 1 Cellular Immunology Laboratory, Biomedical Research Foundation of the Academy of Athens (BRFAA), 11527 Athens, Greece; 2 Department of Thoracic Surgery, Athens Chest Hospital ‘Sotiria’, 11527 Athens, Greece; 3 Molecular Biology Laboratory, BRFAA, 11527 Athens, Greece; 4 Present address: Department of Medicine I, Comprehensive Cancer Center, Institute of Cancer Research, Medical University of Vienna, 1090 Vienna, Austria; 5 Greek Genome Center, BRFAA, 11527 Athens, Greece; 6 Intensive Care Unit, Athens Chest Hospital ‘Sotiria’, 11527 Athens, Greece; 7 7th Respiratory Medicine Department and Asthma Center, Athens Chest Hospital ‘Sotiria’, 11527 Athens, Greece

**Keywords:** Activin-A, Anti-tumor immunity, CD4^+^ T cells, Non-small cell lung cancer, T cell exhaustion, Immune regulation

## Abstract

**Background:**

Although tumor-infiltrating T cells represent a favorable prognostic marker for cancer patients, the majority of these cells are rendered with an exhausted phenotype. Hence, there is an unmet need to identify factors which can reverse this dysfunctional profile and restore their anti-tumorigenic potential. Activin-A is a pleiotropic cytokine, exerting a broad range of pro- or anti-inflammatory functions in different disease contexts, including allergic and autoimmune disorders and cancer. Given that activin-A exhibits a profound effect on CD4^+^ T cells in the airways and is elevated in lung cancer patients, we hypothesized that activin-A can effectively regulate anti-tumor immunity in lung cancer.

**Methods:**

To evaluate the effects of activin-A in the context of lung cancer, we utilized the OVA-expressing Lewis Lung Carcinoma mouse model as well as the B16F10 melanoma model of pulmonary metastases. The therapeutic potential of activin-A-treated lung tumor-infiltrating CD4^+^ T cells was evaluated in adoptive transfer experiments, using CD4^−/−^-tumor bearing mice as recipients. In a reverse approach, we disrupted activin-A signaling on CD4^+^ T cells using an inducible model of CD4^+^ T cell-specific knockout of activin-A type I receptor. RNA-Sequencing analysis was performed to assess the transcriptional signature of these cells and the molecular mechanisms which mediate activin-A’s function. In a translational approach, we validated activin-A’s anti-tumorigenic properties using primary human tumor-infiltrating CD4^+^ T cells from lung cancer patients.

**Results:**

Administration of activin-A in lung tumor-bearing mice attenuated disease progression, an effect associated with heightened ratio of infiltrating effector to regulatory CD4^+^ T cells. Therapeutic transfer of lung tumor-infiltrating activin-A-treated CD4^+^ T cells, delayed tumor progression in *CD4*^−/−^ recipients and enhanced T cell-mediated immunity. CD4^+^ T cells genetically unresponsive to activin-A, failed to elicit effective anti-tumor properties and displayed an exhausted molecular signature governed by the transcription factors Tox and Tox2. Of translational importance, treatment of activin-A on tumor-infiltrating CD4^+^ T cells from lung cancer patients augmented their immunostimulatory capacity towards autologous CD4^+^ and CD8^+^ T cells.

**Conclusions:**

In this study, we introduce activin-A as a novel immunomodulatory factor in the lung tumor microenvironment, which bestows exhausted CD4^+^ T cells with effector properties.

**Supplementary Information:**

The online version contains supplementary material available at 10.1186/s13046-021-02092-5.

## Background

Lung cancer represents the leading cause of cancer-related mortalities and morbidities globally and consists of small cell lung carcinoma and non-small cell lung carcinoma (NSCLC), with the latter comprising 85% of cancer cases [1]. NSCLC exhibits a 5-year survival rate in approximately 10% of patients, with most of the patients presenting metastasis or disease recurrence [2]. Although the selection of the appropriate therapy for NSCLC remains stage dependent, recently-emerged immunotherapies aiming to restore efficient anti-tumor immunity, have become standard treatment modalities [2, 3].

Normally, the immune system holds a crucial role in controlling cancer occurrence as it has the potential to surveil, detect and eradicate tumors [4]. CD4^+^ T cells represent a major component of the adaptive immune system and have emerged as critical controllers of anti-tumor immunity in several types of cancer, including NSCLC [3, 5]. Importantly, CD4^+^ T cells are responsible for priming and expanding tumor-specific CD8^+^ T cells through the delivery of survival signals and maintaining long-term CD8^+^ T cell memory responses [6–8]. Still, in the setting of malignancy, transformed cells often exhibit low immunogenicity and escape immune surveillance by creating an immunosuppressive environment that renders T cells exhausted, thus preventing the establishment of effective anti-tumor immunity [9]. Notably, the density and activation status of tumor-infiltrating T cells represent key prognostic tools for patient response to treatment and survival in several cancer types, including NSCLC [10–13]. Considering the crucial role effector T cells play in tumor control, the identification of factors which can reinvigorate dysfunctional T cells and enhance anti-tumor responses represents an attractive therapeutic approach for lung cancer eradication.

Activin-A is a pleiotropic cytokine, which belongs to the TGF-β superfamily and exerts vital functions in fundamental biological processes [14–16]. A growing body of evidence has highlighted the participation of activin-A in the initiation, propagation and resolution of human diseases, including autoimmune and allergic disorders, viral infections and cancer [16]. Pertinent to lung cancer, circulating activin-A levels are increased in a stage-dependent manner and correlate with poor disease prognosis and metastasis [17–20]. Additionally, *INHBA*, the gene encoding for the βA subunit of activin-A, was discovered as one of the highly-mutated genes in lung adenocarcinoma [21]. Activin-A has demonstrated both pro- and anti-proliferative effects on human lung cancer cell lines, supporting the notion that activin-A exerts cell type-specific responses [19, 22, 23]. Nevertheless, there is a significant gap in our knowledge regarding the role of activin-A in the modulation of T cell-mediated anti-tumor immune responses in the context of lung cancer.

The aim of this study is to decipher the role of activin-A in the regulation of CD4^+^ T cell-directed immune responses in the tumor microenvironment, during the development of murine lung tumors. In a translational approach, we sought to evaluate the effects of activin-A on anti-tumor responses by human lung tumor-infiltrating CD4^+^ T cells from NSCLC patients.

## Materials and methods

### Human subjects

Primary human lung tumor tissue and adjacent lung non-malignant tissue was obtained from NSCLC patients who fulfilled the criteria to undergo surgery for therapeutic purposes at the thoracic surgical clinics of Athens Chest Hospital “Sotiria” (*n* = 38). Information on all donors is given in Supplemental Table 1.

### Mice

C57BL/6 and CD4*Cre*ER^T2+/−^ mice were purchased from The Jackson Laboratory. *Acvr1b*^*fl/fl*^ mice were kindly provided by Dr. P. Bertolino (Inserm - Cancer Research Centre Lyon) [24]. *CD4*^−/−^ mice were purchased from The Jackson Laboratory and kindly provided by Dr. K. Karalis (BRFAA). All mice were maintained at BRFAA’s animal facility and in all experiments 8–10-week-old male mice were used.

### Cell lines

The ovalbumin (OVA) expressing Lewis Lung Carcinoma (LLC-OVA) cell line was kindly provided by Dr. M. Tsoumakidou (Biomedical Sciences Research Center Alexander Fleming) and the B16F10 cell line was kindly provided by Dr. A. Efstratiadis (BRFAA).

### In vivo administration of activin-A in lung tumor models

For the syngeneic lung tumor model and the lung metastasis melanoma model, 5 × 10^5^ LLC-OVA and 2.5 × 10^5^ B16F10 cells were intravenously (i.v.) inoculated, respectively. In both set of experiments 2 μg of recombinant activin-A (r-activin-A, R&D Systems) or PBS was administered intraperitoneally (i.p.) 1 day following cancer cell inoculation and then every 3 days. Mice were euthanized between days 18–22 (LLC-OVA, Supplemental Fig. 1A) and days 14–16 (B16F10).

### In vivo disruption of activin-A signaling in LLC-OVA lung tumor model

CD4*Cre*ER^T2+/−^ mice were crossed to *Acvr1b*^fl/fl^ mice to generate CD4*Cre*ER^T2+/−^/*Acvr1b*^*fl/fl*^ mice (CD4/ALK4-KO), where administration of tamoxifen induces the expression of Cre recombinase under the control of the mouse *Cd4* promoter. For this, 75 mg/kg/dose of tamoxifen (Sigma Aldrich) was i.p. administered to mice on a daily basis for 5 consecutive days. On the 5th day of tamoxifen administration, 5 × 10^5^ LLC-OVA cells were i.v. inoculated. Mice were euthanized between days 16–20 (Supplemental Fig. 3C).

### Chemically-induced lung carcinogenesis model

Mice received i.p. injection of ethyl carbamate (urethane) (1 g/kg body weight) diluted in PBS, on a weekly basis for 10 consecutive weeks [25]. Following a 15-week latency period, after the completion of urethane administration, mice were euthanized (Supplemental Fig. 3E).

### Adoptive T cell transfer in LLC-OVA lung tumor model

5 × 10^5^ LLC-OVA cells were i.v. inoculated of C57BL/6 mice. On day 21, mice were euthanized and lung tumor-infiltrating leukocytes were isolated and cultured in the presence of 50 μg/ml of OVA (Sigma Aldrich) and r-activin-A (50 ng/ml) or PBS for 3 days. After 3 days of culture, CD4^+^ T cells were isolated and i.v. inoculated into LLC-OVA tumor-bearing *CD4*^−/−^ mice (Fig. 3A).

### Human cell cultures

Peripheral blood mononuclear cells (PBMCs) were obtained from NSCLC patients by Histopaque (Sigma Aldrich) density-gradient centrifugation. Total CD4^+^ and CD8^+^ T cells were isolated using commercially available kits (Invitrogen).

Primary lung tumor tissue and adjacent lung non-malignant tissue was obtained from NSCLC patients undergoing surgical resection. Tissues were minced and incubated with 0.14 WÃ¼nsch units/ml Liberase TL (Sigma Aldrich) and 0.1 mg/ml DNase I (Sigma Aldrich) at 37 °C for 1 h. Single-cell suspensions were prepared and the tumor infiltrating leukocyte cell fraction was collected at the interface between 40 and 80% discontinuous percoll gradient. Tumor or healthy lung infiltrating T cells were activated with 2 μg/ml soluble anti-human CD3 (OKT3; Biolegend) and 2 μg/ml anti-human CD28 (CD28.2; Biolegend) in the presence of r-activin-A (50 ng/ml) or PBS for 3–5 days. Activin-A or PBS-treated CD4^+^ T cells were isolated using commercially available kits (ThermoFischer Scientific) and further stimulated with soluble anti-human CD3/CD28 (2 μg/ml both) for 3–5 days. The supernatants of these cultures (referred to as condition media-CM) were collected and either used fresh or stored at -80 °C for further experimentation.

For the measurement of proliferation, CD4^+^ T cells were labelled with CellTrace CFSE (5 μM, Invitrogen), stimulated with soluble anti-human CD3/CD28 (2 μg/ml both) in the presence of 50 ng/ml r-activin-A and cultured for 3–5 days.

For the in vitro suppression assays, activin-A or PBS-treated CD4^+^ T cells isolated from primary lung tumors (effectors) were labelled with CellTracker Red CMTPX (1 μM, Invitrogen) and co-cultured with CellTrace CFSE (5 μm, Invitrogen) labelled-CD4^+^ T cells (responders) isolated from peripheral blood at a 2:1 ratio (responders:effectors) in the presence of soluble anti-human CD3 (2 μg/ml; OKT3; Biolegend) and soluble anti-human CD28 (2 μg/ml; CD28.2; Biolegend). The proliferation of responder cells was assessed on days 3–5 via flow cytometry.

For cytotoxicity assays, untouched human CD8^+^ T cells isolated from PBMCs using commercially available kits (ThermoFischer Scientific), were pre-treated for 14-16 h with CM from CD4^+^ T cell cultures of the same patient. In parallel, single cell suspensions collected after the mechanical and enzymatic digestion of primary lung tumors were subjected to negative selection of cancer cells by depleting the CD45^+^ cell fraction. The following day, human CD8^+^ T cells were co-cultured with primary lung cancer cells at different ratios (5:1–40:1) for 4-6 h and CD8^+^ T cell mediated cytotoxicity was quantitated by measuring the activation of Caspase-3 in cancer cells via flow cytometry and the release of LDH in the culture supernatants using the LDH Cytotoxicity Detection Kit (Takara). For the assessment of Caspase-3 activation, cells were stained with antibody against human CD8 (Biolegend) and then incubated with 2.5 μM NucView 488 Caspase-3 substrate (Biotium) at RT for 30 min.

### Murine cell cultures

Lung tissues from mice bearing either LLC-OVA lung tumors or B16F10 melanoma lung metastases were collected and minced and incubated with 0.14 WÃ¼nsch units/ml Liberase TL (Sigma Aldrich) and 0.1 mg/ml DNase I (Sigma Aldrich) at 37 °C for 1 h. Single-cell suspensions were prepared and the tumor-infiltrating leukocyte cell fraction was collected at the interface between 40 and 80% discontinuous percoll gradient. Activation of tumor-infiltrating T cells from LLC-OVA tumor-bearing mice was induced with the addition of 50 μg/ml OVA in the presence of r-activin-A (50 ng/ml) or PBS for 3–5 days.

For the measurement of proliferation, CD4^+^ T cells were isolated from the tumor-infiltrating leukocyte fraction using commercially available kits (Biolegend), labelled with CellTrace CFSE (5 μM, Invitrogen) and cultured in the presence of 50 μg/ml OVA and mitomycin-C-treated (Sigma Aldrich) splenic APCs for 4 days. CM were collected and stored at -80 °C for further experimentation and CD4^+^ T cell proliferation was measured via flow cytometry.

For cytotoxicity assays, LLC-OVA cells were seeded in different densities and the following day, CD8^+^ T cells isolated from the lungs of tumor-bearing mice were co-cultured with LLC-OVA cells at different ratios for 4–6 h. CD8^+^ T cell mediated cytotoxicity was quantitated by measuring the activation of Caspase-3 in LLC-OVA cells via flow cytometry and by measuring the release of LDH in the culture supernatants using the LDH Cytotoxicity Detection Kit (Takara). For the assessment of Caspase-3 activation, cells were stained with antibody against mouse CD8 (Biolegend) and then incubated with 2.5 μM NucView 488 Caspase-3 substrate (Biotium) at RT for 30 min.

### Lung histology

Paraffin-embedded sections (4 μm) were stained with hematoxylin & eosin (H&E) to evaluate lung infiltration, as described previously [26].

### Confocal microscopy

For immunofluorescence experiments, lung sections were deparafinized, rehydrated and blocked in 10% donkey serum, diluted in 0.1% Triton-X. Lung sections were incubated overnight at 4 °C with a primary antibody against mouse/human activin-A or isotype controls (R&D Systems). Lung sections were incubated with fluorescently-labelled secondary antibodies (Invitrogen) for 1 h at room temperature (RT). Nuclear staining and mounting were carried out using DAPI (ThermoFischer Scientific) and Fluoromount-G (eBioscience). Image acquisition was performed using a confocal laser scanning microscope (Leica TCS SP5). Immunofluorescence data analysis was performed with the Image J software.

### Cytokine analysis

Cytokines were measured in murine and human cell culture supernatants and mouse lung homogenates using commercially available ELISA kits for mouse and human IFN-γ, TNF-α, IL-2, IL-10 and activin-A (R&D Systems).

### Flow-cytometry analysis

Cells were stained with fluorescently-labelled antibodies to mouse CD45, CD3, CD4, CD8 (Thermo Scientific), PD-1, CTLA-4, LAG-3, Tim-3, perforin, granzyme-b (Biolegend), Tox/Tox2 (Cell signaling) and to human CD45, CD4, CD69 (ThermosScientific), PD-1, CTLA-4 and LAG-3 (Biolegend). For intracellular cytokine staining the Cyto-Fast Fix/Perm Buffer Set (Biolegend) was used. Cells were re-stimulated with PMA (20 ng/ml)/ionomycin (500 ng/ml) (Sigma-Aldrich) and Golgi-Stop (1 μl/ml) (BD Biosciences) and stained with antibodies against mouse or human IL-10 and IFN-γ (eBiosciences). Foxp3 and Tox/Tox2 staining was performed using the True-Nuclear Transcription Factor Buffer Set (Biolegend). FACS acquisition was performed with the cytometer Cytomics FC500 (Beckman Coulter) and a BD FACSAria II (Becton Dickinson) and the data were analyzed using the FlowJo X software 10.07 (Tree Star, Inc).

### Western blot analysis

CD4^+^ T cells (1 × 10^6^) were isolated from the lung tumors of CD4/ALK4-KO or WT mice. Cells were washed with PBS and harvested in lysis buffer supplemented with protease and phosphatase inhibitors (Invitrogen). Protein homogenates were loaded on acrylamide gels (Biorad), transferred onto a PVDF membrane (Millipore), blocked with 5% non-fat milk (or 5% BSA for phosphor-mTOR) at 25oC for 1 h, and probed with primary antibodies against phosphor-mTOR (Cell Signaling), TATA-binding protein (TBP) (Santa Cruz), Tox/Tox2 (Cell signaling) and GAPDH (Merck Millipore), at 4oC overnight. The blot was subsequently incubated with horseradish peroxidase-linked secondary antibodies at 25oC for 1 h, and developed using the Immobilon Forte Western HRP substrate (Merck Millipore).

### RNA-sequencing analysis

RNA-Sequencing (RNA-Seq) libraries were prepared with the illumina TruSeq RNA v2 kit using 1 μg of total RNA, checked with the Agilent bioanalyzer DNA1000 chip, quantitated with the qubit HS spectrophotometric method and pooled in equimolar amounts for Sequencing. Approximately 25Million, 75 bp long, Single-End reads were generated for each sample with illumina NextSeq500 sequencer.

Quality Control was performed at the fastq raw data file for each sample using the “FASTQC” software. FastQ files were aligned to mm10 mouse genome using HISAT2 [27]. Counts were defined using HTSeq htseq-count command with the “intersection non empty” option [28]. The count files were used as Input for DESeq2 [29]. Normalization was performed with the estimate size factor function followed by Differentially Expressed Genes Analysis. For Principle Component Analysis, normalization was performed with rlog function to log2 scale. Pathway analysis was performed at EnrichR [30, 31]. Data for Pathway analysis were retrieved from Molecular Signature Database [32]. DEGs Volcano Plot and Pathway Dotplot was created in R with ggplot2 package. Heatmaps were constructed with pheatmap package in R after computing the respective z-score. All gene sets derive from Molecular Signature Database except for glutathione gene set, which originates from KEGG 2019 [33]. Pearson correlation coefficient analysis was performed in R with ggplot2 and ggpubr package from data retrieved from Page et al., 2018 (GSE93804).

Gene Set Enrichment Analysis was performed from gene sets originating from immunologic gene signature database with WebGestallt package in R [34, 35].

### Chromatin immunoprecipitation

CD4^+^ T cells (8 × 10^6^) were isolated from the lung tumors of CD4/ALK4-KO or WT mice and chromatin immunoprecipitation (ChIP) was performed using the SimpleChIP Plus Enzymatic Chromatin IP Kit (Cell Signaling Technology). Chromatin fragments were incubated overnight at 4 °C with anti-Tox/Tox2 (Cell Signaling Technology) or normal rabbit IgG (Cell Signaling Technology), as a negative control. The relative enrichment of ChIP versus IgG (relative to input DNA) precipitated DNA was determined by qPCR analysis for the regions of interest, using the following primer pairs:

*Pdcd1*: FW: 5′- ACCTTTCCTGTGCCTACGTC - 3′, REV: 5′ - TAAGAGTGGTGGTGGTTGGG - 3′ [36];.

*Lag3*: FW: 5′- CTCCTCCAGACCCAGTCCTT - 3′, REV: 5′ - GAATCAGCCCCCTCACACTT - 3′; *Tigit*: FW: 5′- GATGTGGCCTCTCCTCCAAC - 3′, REV: 5′ - GGCTCACCCTATGTGGCTTT - 3′; *Nfatc1*: FW: 5′- CCGCAAAGTTTCTCCGCTC - 3′, REV: 5′ - GCGTGGATGCCAAGTACCA - 3′; *Jun*: FW: 5′- AAGTTGCTGAGGTTGGCGTA - 3′, REV: 5′ - GGCTGAACTGCATAGCCAGA - 3′; *Txn1*: FW: 5′- GGAGCCAGAGTCGGCTACTA - 3′, REV: 5′ - TGTTCGGCAGCTTAGAGTGG - 3′.

Primer sets were designed near Tox binding regions according to published ChIP-Sequencing (ChIP-Seq) data (GSE93953) [37].

### Statistical analysis

Graph Pad Prism 8 (Graph Pad Software Inc.) was used for all statistical analyses. All data are presented as mean ± SEM. The comparison of survival curves was performed using Gehan-Breslow-Wilcoxon test. To calculate differences between groups, we used Student’s t test, the Mann-Whitney test and the Wilcoxon matched-pairs signed rank test as appropriate. We considered any difference with a *p* value of 0.05 or less to be statistically significant (*p ≤ 0.05, **p ≤ 0.01 and ***p ≤ 0.001).

## Results

### In vivo administration of activin-A attenuates the development and progression of lung tumors

**Fig. 1:**
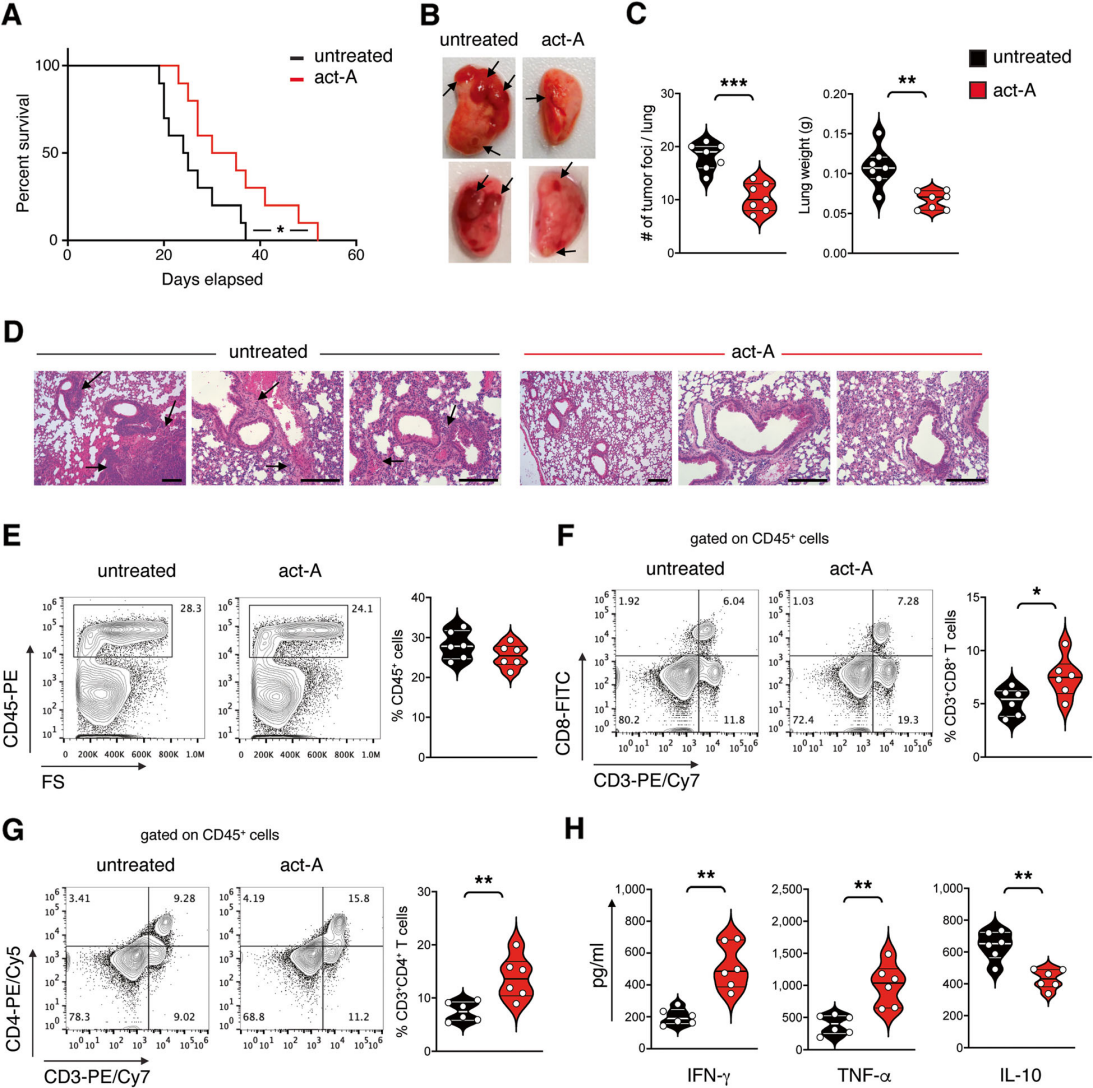
In vivo administration of activin-A attenuates the development and progression of lung tumors. **A** Survival plot of activin-A-treated or untreated LLC-OVA-tumor bearing mice (*n* = 10). **B** Representative macroscopic images of LLC-OVA-tumor bearing lung lobes. **C** Tumor foci and lung weight measurements of activin-A-treated or untreated LLC-OVA-tumor bearing mice (*n* = 7). **D** Representative photomicrographs of H&E-stained lung sections (scale bars: 100 μm). **E** Representative flow cytometry plots (left) and cumulative percentages of CD45^+^ lung tumor-infiltrating cells (right). **F** Representative flow cytometry plots (left) and cumulative percentages of CD3^+^CD8^+^ T cells among CD45^+^ lung tumor-infiltrating cells (right). **G** Representative flow cytometry plots (left) and cumulative percentages of CD3^+^CD4^+^ T cells among CD45^+^ lung tumor-infiltrating cells (right). **H** Cytokine levels in supernatants of lung tumor-infiltrating leukocyte cultures. Data are mean ± SEM of triplicate wells. Data are representative of 6 independent experiments. Statistical significance was obtained by Log-rank (Mantel-Cox) test and unpaired Student’s t-test; *P < 0.05 and **P < 0.01

We initially examined the in vivo role of activin-A in the establishment of T-cell mediated anti-tumor immune responses in the airways using the syngeneic LLC-OVA mouse lung cancer model (Supplemental Fig. 1A). We observed that activin-A treatment of lung cancer-bearing mice (act-A-treated) led to prolonged overall survival, accompanied by delayed disease onset compared to mice that received PBS (untreated) (Fig. 1A). Additionally, act-A-treated mice developed fewer and smaller superficial lung tumors, and as a result, displayed reduced lung weight compared to untreated mice (Fig. 1B-C). H&E staining demonstrated that act-A-treated mice showed delayed development of peribronchial and perivascular tumors which were clearly formed in the PBS group (Fig. 1D). To address whether the reduction in tumor growth was attributed to enhanced anti-tumor responses, we evaluated intra-tumoral infiltration by leukocytes. We did not observe any differences in total CD45^+^ tumor-infiltrating leukocytes between act-A-treated and untreated mice (Fig. 1E). Nevertheless, the percentages of CD8^+^ and mostly CD4^+^ T cells infiltrating the lungs were significantly elevated in act-A-treated mice compared to untreated mice (Fig. 1F-G). In contrast, the percentages of several other subsets of myeloid origin, such as, F4/80^+^ macrophages, CD11b^+^ myeloid cells and CD11c^+^ dendritic cells (DCs) that infiltrated lung tumors were marginally lower in act-A-treated mice (Supplemental Fig. 1B). Importantly, the infiltration of granulocytic (CD11b^+^Ly6G^+^) and monocytic (CD11b^+^Ly6C^+^) myeloid derived suppressor cells was significantly decreased in act-A-treated mice (Supplemental Fig. 1C). Notably, tumor-infiltrating lymphocytes (TILs) isolated from act-A-treated mice secreted higher levels of the typical effector cytokines IFN-γ and TNF-α and lower levels of the immunosuppressive cytokine IL-10 upon ex vivo antigenic stimulation compared to untreated mice. (Fig. 1H). We further evaluated the effects of activin-A in a metastatic B16F10 model of melanoma in the lungs. Interestingly, activin-A treatment led to prolonged overall survival (Supplemental Fig. 1D) and impaired formation of melanoma metastases in the lungs of B16F10-inoculated mice (Supplemental Fig. 1E-F). Although the infiltration of lung tumors with CD8^+^ T cells was unaltered by activin-A administration, CD4^+^ T cell infiltration was enhanced in act-A-treated mice (Supplemental Fig. 1G-H). Collectively, our findings provide evidence that activin-A restrains the formation of primary and metastatic tumors in the lungs through the enhancement of anti-tumor T effector responses.

### Activin-A represses the expression of inhibitory receptors on lung tumor-infiltrating CD4^+^ T cells

**Fig. 2:**
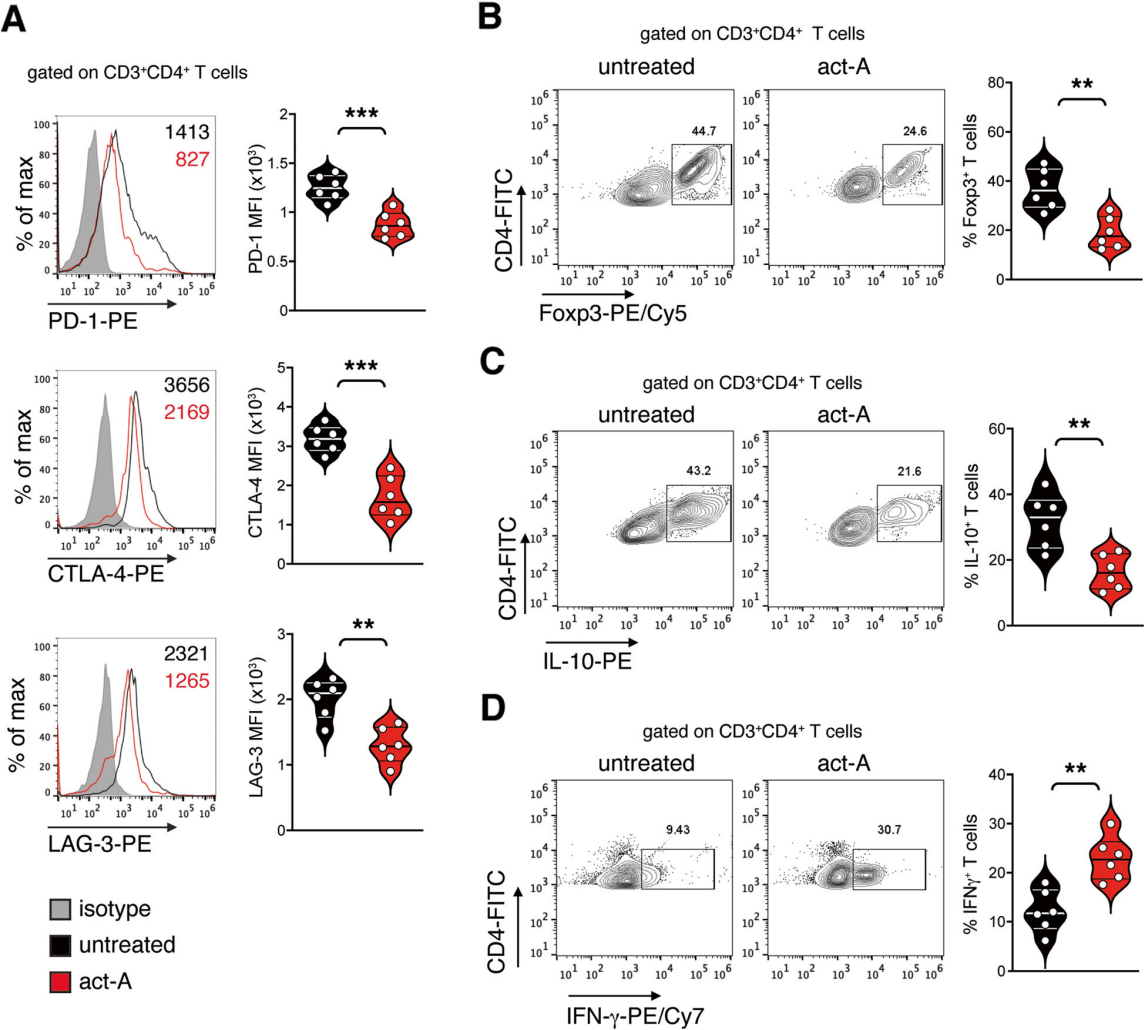
Activin-A represses the expression of inhibitory receptors on lung tumor-infiltrating CD4^+^ T cells. **A** Representative flow cytometry plots (left) and cumulative median fluorescence intensity (MFI) values of PD-1, CTLA-4 or LAG-3 expression by CD3^+^CD4^+^ T cells infiltrating lung tumors (right) from activin-A-treated or untreated LLC-OVA-tumor bearing mice. Numbers in plots indicate MFI values. Shaded histograms represent isotype controls. **B** Representative flow cytometry plots (left) and cumulative percentages of Foxp3-expressing cells among CD3^+^CD4^+^ T cells infiltrating lung tumors (right). **C** Representative flow cytometry plots (left) and cumulative percentages of IL-10-expressing cells among CD3^+^CD4^+^ T cells infiltrating lung tumors (right). **D** Representative flow cytometry plots (left) and cumulative percentages of IFN-γ-expressing cells among CD3^+^CD4^+^ T cells infiltrating lung tumors (right). Data are representative of 6 independent experiments. Statistical significance was obtained by unpaired Student’s t-test; **P < 0.01 and ***P < 0.001

Taking into consideration that activin-A administration in vivo considerably enhanced the infiltration of CD4^+^ T cells in the lungs of tumor-bearing mice, we next sought to characterize the profile of these cells. We discovered that CD3^+^CD4^+^ T cells infiltrating the lungs of act-A-treated LLC-OVA-bearing mice expressed lower levels of the hallmark inhibitory immune receptors PD-1, CTLA-4 and LAG-3 compared to untreated mice (Fig. 2A). Notably, we also observed significantly fewer Foxp3-expressing regulatory CD4^+^ T cells infiltrating the lung tumors of act-A-treated mice compared to untreated mice (Fig. 2B). Moreover, we detected heightened percentages of IFN-γ-producing CD4^+^ T cells among TILs, concomitantly with decreased numbers of IL-10-producing CD4^+^ T cells in act-A-treated mice compared to untreated mice (Fig. 2C-D). In line with the aforementioned results, CD4^+^ T cells infiltrating the lungs of act-A-treated, B16F10-inoculated mice expressed less CTLA-4 and LAG-3 and slightly lower levels of PD-1 (Supplemental Fig. 2A). Once more, in this model, we observed significantly fewer Foxp3-expressing regulatory CD4^+^ T cells accompanied by less IL-10-producing and marginally more IFN-γ-expressing CD4^+^ T cells infiltrating lung metastases in act-A-treated mice compared to untreated mice (Supplemental Fig. 2B-C). Overall, our data suggest that activin-A-mediated lung tumor regression is associated with increased numbers of tumor-infiltrating CD4^+^ T cells with effector phenotype at the expense of CD4^+^ T cells with suppressive properties.

### Therapeutic transfer of activin-A-exposed CD4^+^ T cells decelerates the progression of lung tumor formation

**Fig. 3:**
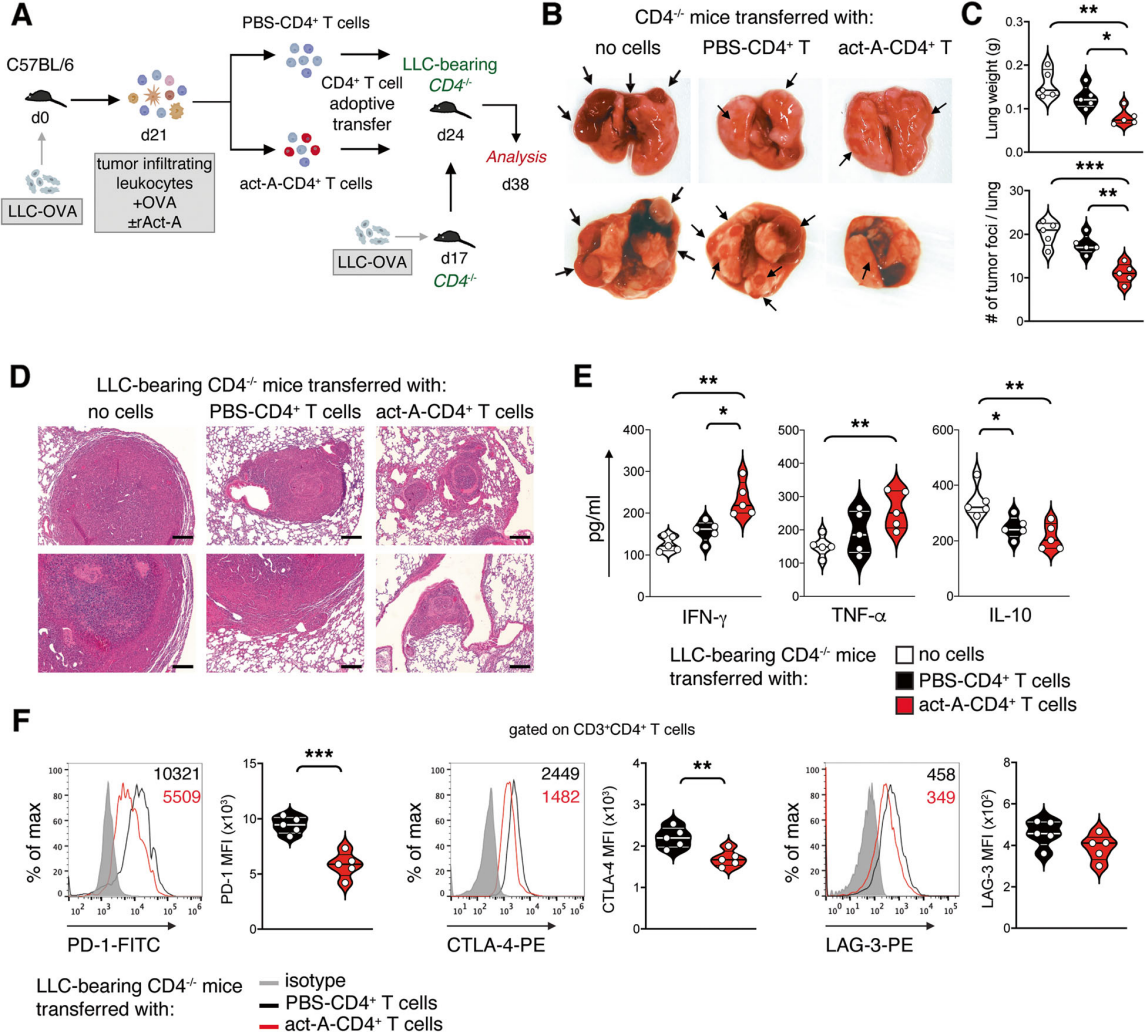
Therapeutic transfer of activin-A-exposed CD4^+^ T cells decelerates the progression of lung tumor formation. **A** Experimental protocol utilized. **B** Representative macroscopic images of lung lobes of LLC-OVA-tumor bearing *CD4*^−/−^ mice adoptively transferred with ex vivo activin-A- or PBS-treated CD4^+^ T cells isolated from lung tumors of LLC-OVA-inoculated C57BL/6 mice. **C** Lung weight and tumor foci measurements. **D** Representative photomicrographs of H&E-stained lung sections (scale bars: 50 μm). **E** Cytokine levels in supernatants of lung tumor-infiltrating leukocyte cultures. Data are mean ± SEM of triplicate wells and are representative of 5 independent experiments. **F** Representative flow cytometry plots (left) and cumulative MFI values of PD-1, CTLA-4 or LAG-3 expression by CD3^+^CD4^+^ T cells infiltrating lung tumors (right). Data are mean ± SEM and are representative of 5 independent experiments. Statistical significance was obtained by unpaired Student’s t-test; *P < 0.05, **P < 0.01 and ***P < 0.001

On the basis of these findings, we assessed whether adoptive transfer of lung tumor-infiltrating CD4^+^ T cells treated ex vivo with activin-A could reduce lung tumor burden and enhance the overall survival of LLC-OVA tumor-bearing mice (Fig. 3A). Notably, adoptive transfer of activin-A-treated CD4^+^ T cells (act-A-CD4^+^ T cells) into tumor-bearing *CD4*^−/−^ mice led to restrained lung tumor formation in comparison to mice transferred with PBS-treated CD4^+^ T cells (PBS-CD4^+^ T cells) or mock-transferred *CD4*^−/−^ mice (no cells) (Fig. 3B-D). In addition, IFN-γ and TNF-α levels were significantly upregulated in culture supernatants of TILs isolated from the lungs of act-A-CD4^+^ T cell-transferred *CD4*^−/−^ mice, upon antigenic re-stimulation, compared to PBS-CD4^+^ T cell-transferred *CD4*^−/−^ mice and to greater extent to mock-transferred *CD4*^−/−^ mice (Fig. 3E). Concomitantly, the secretion of IL-10 in culture supernatants of TILs isolated from act-A-CD4^+^ T cell-transferred *CD4*^−/−^ mice was considerably decreased in comparison to PBS-CD4^+^ T cell- and mock-transferred *CD4*^−/−^ mice (Fig. 3E). Notably, act-A-CD4^+^ T cells maintained a less exhausted phenotype in vivo relatively to PBS-CD4^+^ T cells, as evidenced by strongly decreased levels of PD-1 and CTLA-4 (Fig. 3F). Overall, our data demonstrate that therapeutic transfer of ex vivo-generated act-A-CD4^+^ T cells to LLC-OVA-bearing *CD4*^−/−^ mice leads to a marked reduction in tumor burden of recipients and protects against disease progression.

### Disruption of activin-A signaling in CD4^+^ T cells accelerates lung tumor progression and alters the profile of tumor-infiltrating CD4^+^ T cells

**Fig. 4:**
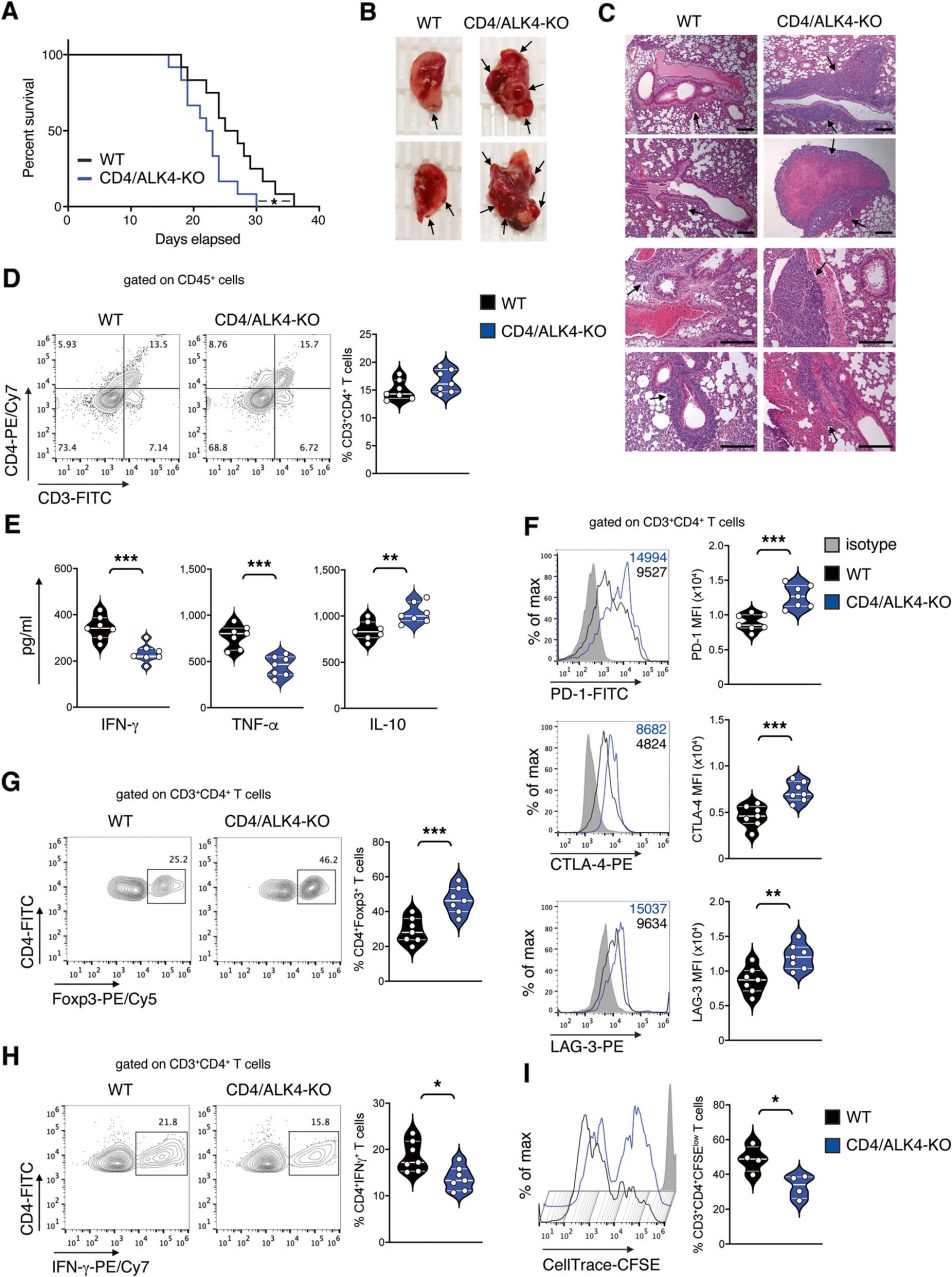
Disruption of activin-A signaling in CD4^+^ T cells accelerates lung tumor progression and alters the profile of tumor-infiltrating CD4^+^ T cells. **A** Survival plot of CD4/ALK4-KO or WT LLC-OVA-tumor bearing mice (*n* = 12). **B** Representative macroscopic images of lung lobes. **C** Representative photomicrographs of H&E-stained lung sections (scale bars: 100 μm). **D** Representative flow cytometry plots (left) and percentages of CD3^+^CD4^+^ T cells among CD45^+^ lung tumor-infiltrating cells (right). **E** Cytokine levels in supernatants of lung tumor-infiltrating CD4^+^ T cells. Data are mean ± SEM of triplicate wells. **F** Representative flow cytometry plots (left) and cumulative MFI values of PD-1, CTLA-4 or LAG-3 expression by CD3^+^CD4^+^ T cells infiltrating lung tumors (right). **G** Representative flow cytometry plots (left) and cumulative percentages of Foxp3-expressing cells among CD3^+^CD4^+^ T cells infiltrating lung tumors (right). **H** Representative flow cytometry plots (left) and cumulative percentages of IFN-γ-expressing cells among CD3^+^CD4^+^ T cells infiltrating lung tumors (right). **I** Representative flow cytometry plot (left) and cumulative percentages of CD3^+^CD4^+^CFSE^low^ T cells isolated from lung tumors (right). Data are mean ± SEM and are representative of 7 (**D-H**) and 4 (**I**) independent experiments. Statistical significance was obtained by Log-rank (Mantel-Cox) test and unpaired Student’s t-test; *P < 0.05, **P < 0.01 and ***P < 0.001

Several studies have shown that activin-A expression is increased in the serum and biopsies of lung cancer patients [17–20]. In accordance with the human studies, we also observed elevated activin-A levels in the serum and lungs of LLC-OVA-bearing mice (Supplemental Fig. 3A-B). Since activin-A was overexpressed during lung tumor formation in the LLC-OVA model and displayed a profound effect on lung tumor-infiltrating CD4^+^ T cells, we subsequently examined the effects of endogenous activin-A specifically on CD4^+^ T cells. For this purpose, we utilized an inducible model of CD4^+^ T cell-specific knockout of ALK4, activin-A’s major type I signaling receptor (CD4/ALK4-KO) (Supplemental Fig. 3C). Initially, LLC-OVA-inoculated CD4/ALK4-KO mice exhibited decreased overall survival, accompanied by the development of larger lung tumors, as evidenced by macroscopic observations, lung weights evaluation and H&E staining assessment (Fig. 4A-C, Supplemental Fig. 3D) compared to their wild-type littermates (WT). We further verified the anti-tumorigenic effect of activin-A signaling on CD4^+^ T cells, in a physiologically relevant chemically-induced model of lung cancer (Supplemental Fig. 3E) [25]. In line with the aforementioned findings, we observed that urethane-treated CD4/ALK4-KO mice exhibited increased lung tumor formation compared to their WT counterparts (Supplemental Fig. 3F-G).

Disruption of activin-A signaling in CD4^+^ T cells had no impact on either total CD45^+^ leukocytes nor CD3^+^CD8^+^ and CD3^+^CD4^+^ T cells infiltrating the lung tumors of LLC-OVA-bearing mice (Fig. 4D, Supplemental Fig. 3H-I). Nevertheless, we found that CD4^+^ T cells isolated from the lung tumors of CD4/ALK4-KO mice secreted significantly less IFN-γ and TNF-α concomitantly with increased levels of IL-10 upon ex vivo antigenic re-stimulation compared to CD4^+^ T cells from WT mice, suggesting that ablation of activin-A signaling specifically in CD4^+^ T cells restrains the development of effective anti-tumor T cell responses (Fig. 4E). These findings were further strengthened by the observations that CD4^+^ T cells from lung tumors of CD4/ALK4-KO mice exhibited a more exhausted profile as exemplified by higher levels of PD-1, CTLA-4 and LAG-3 compared to WT mice (Fig. 4F). In addition, we observed significantly increased percentages of Foxp3-expressing regulatory T cells concomitantly with decreased frequencies of IFN-γ-expressing CD4^+^ T cells infiltrating the lung tumors of CD4/ALK4-KO mice compared to WT mice (Fig. 4G-H). In order to more precisely assess the exhaustion profile of CD4^+^ T cells infiltrating the lung tumors, we evaluated the expression of PD-1, CTLA-4 and LAG-3 by CD4^+^Foxp3^−^ T cells, since regulatory T cells are known to increase the production of multiple inhibitory receptors. We detected higher expression of the latter by CD4^+^ Foxp3^−^ T cells from lung tumors of CD4/ALK4-KO mice compared to WT mice (Supplemental Fig. 4A). On the contrary, upon activin-A administration in vivo, CD4^+^Foxp3^−^ T cells infiltrating the lungs of act-A-treated LLC-OVA-bearing mice expressed lower levels of PD-1, CTLA-4 and LAG-3 compared to untreated mice (Supplemental Fig. 4B). Importantly, CD4^+^ T cells from CD4/ALK4-KO mice appeared to be less capable to proliferate in response to antigenic stimulation in comparison to CD4^+^ T cells from WT mice (Fig. 4I). Taken together, the aforementioned results suggest that disruption of activin-A signaling in CD4^+^ T cells significantly deteriorates anti-tumor immunity and augments lung tumor growth in vivo.

### Ablation of activin-A signaling in CD4^+^ T cells alters the profile and attenuates the effector function of lung tumor-infiltrating CD8^+^ T cells

**Fig. 5:**
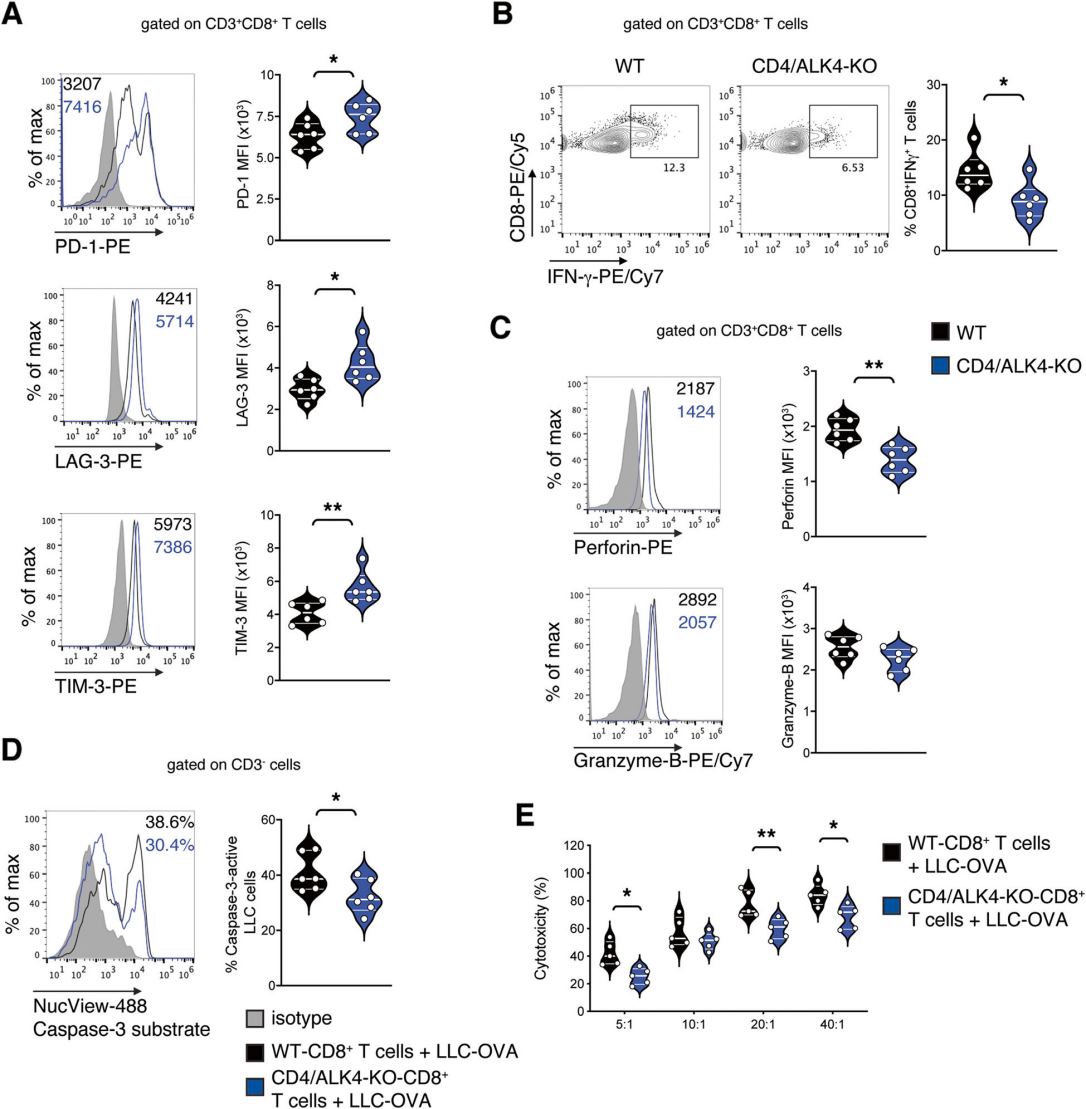
Ablation of activin-A signaling in CD4^+^ T cells alters the profile and attenuates the effector function of lung tumor-infiltrating CD8^+^ T cells. **A** Representative flow cytometry plots (left) and cumulative MFI values of PD-1, LAG-3 or TIM-3 expression by CD3^+^CD8^+^ T cells infiltrating lung tumors of CD4/ALK4-KO or WT LLC-OVA-tumor bearing mice (right). **B** Representative flow cytometry plots (left) and cumulative percentages of IFN-γ-expressing cells among CD3^+^CD8^+^ T cells infiltrating lung tumors (right). **C** Representative flow cytometry plots (left) and cumulative MFI values of perforin and granzyme-B expression by CD3^+^CD8^+^ T cells infiltrating lung tumors (right). **D** LLC-OVA cells were co-cultured with lung tumor-infiltrating CD8^+^ T cells from CD4/ALK4-KO or WT LLC-OVA-tumor bearing mice. Representative flow cytometry plot (left) and cumulative percentages (right) depicting caspase-3 activity of LLC-OVA cells. **E** LLC-OVA cells were co-cultured (at indicated ratios) with lung tumor-infiltrating CD8^+^ T cells from CD4/ALK4-KO or WT LLC-OVA-tumor bearing mice. Cytotoxicity was assessed by LDH measurement in cell culture supernatants. Data are mean ± SEM and are representative of 6 (**A-D**) and 2 (**E**) independent experiments. Statistical significance was obtained by unpaired Student’s t-test; *P < 0.05 and **P < 0.01

Considering the crucial role that CD8^+^ T cells play in tumor eradication [38, 39], we next interrogated whether CD4^+^ T cells that are unresponsive to activin-A signaling in vivo alter the phenotype and activation status of CD8^+^ T cells infiltrating lung tumors of CD4/ALK4-KO mice compared to their WT counterparts. Markedly, CD8^+^ T cells that infiltrated the lung tumors of CD4/ALK4-KO mice demonstrated higher expression of PD-1, LAG-3 and TIM-3, in comparison to CD8^+^ T cells obtained from WT mice, suggesting that these cells had also acquired a more exhausted phenotype (Fig. 5A). Moreover, CD8^+^ T cells from CD4/ALK4-KO mice were characterized by reduced expression of effector molecules, such as IFN-γ, perforin and, to less extent, granzyme-B (Fig. 5B-C). In view of these findings, we next examined the cytotoxic capacity of CD8^+^ T cells infiltrating the lung tumors of CD4/ALK4-KO and WT mice. Interestingly, CD8^+^ T cells from CD4/ALK4-KO mice exhibited diminished cytotoxic potential, as shown by reduced induction of apoptosis of the targeted LLC-OVA cells as compared to CD8^+^ T cells from WT mice (Fig. 5D-E). Overall, our data propose that CD4^+^ T cells that are unresponsive to activin-A signaling in vivo modulate the effector properties and cytotoxic capacity of CD8^+^ T cells infiltrating the lung tumors of CD4/ALK4-KO mice, further contributing to disease progression.

### Activin-A-treated human lung tumor-infiltrating CD4^+^ T cells exhibit a less exhausted profile

**Fig. 6:**
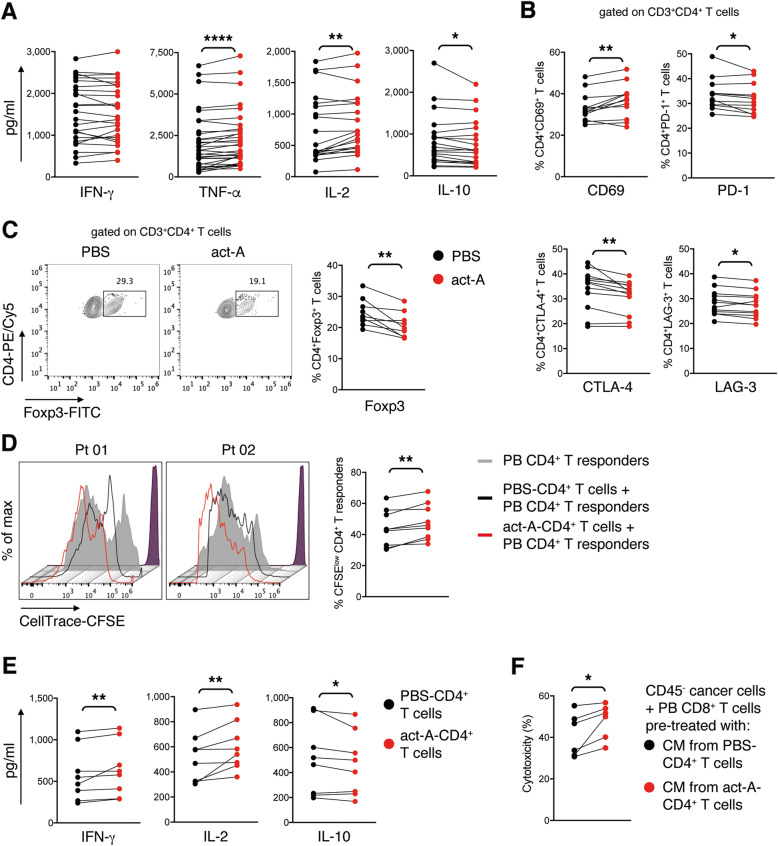
Activin-A-treated human lung tumor-infiltrating CD4^+^ T cells exhibit a less exhausted profile. **A** Primary lung tumor-infiltrating leukocytes were isolated from NSCLC patients and stimulated with antibodies against CD3/CD28 in the presence of activin-A or PBS. Cytokine levels in culture supernatants of PBS- or activin-A-treated lung tumor-infiltrating leukocytes (*n* = 20–25 donors). Data are mean ± SEM of triplicate wells. **B** Cumulative percentages of CD69, PD-1, CTLA-4 or LAG-3-expressing cells among human lung tumor-infiltrating CD3^+^CD4^+^ T cells cultured as in Fig. 6A (*n* = 12 donors). **C** Representative flow cytometry plots (left) and cumulative percentages (right) of Foxp3-expressing cells among human lung tumor-infiltrating CD3^+^CD4^+^ T cells cultured as in Fig. 6A (*n* = 9 donors). **D** Activin-A- or PBS-treated human lung tumor-infiltrating CD4^+^ T cells, generated as in Fig. 6A, were CellTracker Red-labelled and co-cultured with CellTrace-CFSE-labelled autologous peripheral blood (PB) CD4^+^ T responders. Representative flow cytometry plots showing T responder cell proliferation (left) and cumulative data (right) (*n* = 9 donors). **E** Cytokine levels in culture supernatants of lung tumor-infiltrating act-A- or PBS-CD4^+^ T cells (*n* = 8 donors). Data are mean ± SEM of triplicate wells. **F** CD45^−^ lung cancer cells were co-cultured with autologous PB CD8^+^ T cells pre-treated with CM from act-A- or PBS-CD4^+^ T cell cultures. Cumulative data of cytotoxicity, assessed by LDH measurement in cell culture supernatants (*n* = 6). Statistical significance was obtained by Wilcoxon matched-pairs signed rank test; *P < 0.05, **P < 0.01 and ****P < 0.0001

A major caveat in the use of preclinical mouse models in most types of cancer is that they fail to recapitulate the complexity of the malignancy, as well as, the immune contexture within the tumor microenvironment [40]. For this, we next explored the potential anti-tumorigenic effects of activin-A on T cells from treatment-naïve patients with NSCLC (Supplemental Table 1). We observed increased expression of the effector cytokines TNF-α and IL-2 concomitantly with decreased secretion of IL-10 in culture supernatants of tumor-infiltrating leukocytes treated ex vivo with activin-A (Fig. 6A). Interestingly, culture supernatants from adjacent non-malignant lung leukocytes exhibited reduced levels of IFN-γ and IL-2 upon ex vivo activin-A administration, indicating that activin-A exerts distinct effects on T cells that infiltrate the lung tumors compared to those found in the adjacent non-malignant lung tissue (Supplemental Fig. 5A). We then characterized the profile of human CD4^+^ T cells infiltrating the lung tumors, upon ex vivo activin-A treatment. Notably, we observed that activin-A-treated CD4^+^ T cells (act-A-CD4^+^ T cells) displayed heightened expression of the activation marker CD69, concomitantly with reduced expression of the immune checkpoint inhibitory molecules PD-1, CTLA-4 and LAG-3 compared to PBS-treated CD4^+^ T cells (PBS-CD4^+^ T cells) (Fig. 6B). In line with the aforementioned results, act-A-CD4^+^ T cells demonstrated reduced expression of the transcription factor Foxp3 (Fig. 6C), suggesting that activin-A endows human lung tumor-infiltrating CD4^+^ T cells with a less exhausted phenotype. Despite the fact that act-A-CD4^+^ T cells seemed to have acquired a more activated phenotype compared to PBS-CD4^+^ T cells, both populations exhibited similar capacity to proliferate upon ex vivo polyclonal stimulation (Supplemental Fig. 5B).

Next, in an attempt to evaluate the immunostimulatory capacity of tumor-infiltrating CD4^+^ T cells exposed to activin-A in vitro, we cultured them along with autologous CD4^+^ T cells obtained from the periphery of the same patient (PB CD4^+^ T cells) upon polyclonal stimulation. We observed that PB CD4^+^ T responder cells cultured along with act-A-CD4^+^ T cells demonstrated increased proliferation compared to those cultured with PBS-CD4^+^ T cells (Fig. 6D). This finding was complemented by the observation that tumor-infiltrating act-A-CD4^+^ T cells secreted heightened levels of IFN-γ and IL-2 and lower levels of the immunosuppressive cytokine IL-10 upon polyclonal stimulation (Fig. 6E). We next sought to investigate whether act-A-CD4^+^ T cells could also directly affect the cytotoxic capacity of autologous PB CD8^+^ T cells. For this purpose, condition medium (CM) from tumor-infiltrating act-A- or PBS-CD4^+^ T cell cultures was supplemented into PB CD8^+^ T cells. Subsequently, PB CD8^+^ T cells supplemented with CM from tumor-infiltrating act-A- or PBS-CD4^+^ T cell cultures were co-cultured with autologous CD45^−^ lung cancer cells. Remarkably, we discovered that PB CD8^+^ T cells supplemented with CM from act-A-CD4^+^ T cell cultures were more competent in killing the targeted primary lung cancer cells, compared to PB CD8^+^ T cells supplemented with CM from PBS-CD4^+^ T cell cultures (Fig. 6F, Supplemental Fig. 5C). Overall, in a translational approach, we demonstrate that ex vivo activin-A treatment endows human lung tumor-infiltrating CD4^+^ T cells with a less exhausted phenotype and concomitantly boosts their immunostimulatory capacity towards autologous CD4^+^ and CD8^+^ T cells, mimicking its anti-tumorigenic effects observed in the mouse lung cancer models.

### Ablation of activin-A signaling renders CD4^+^ T cells exhausted via a Tox/Tox2-dictated transcriptional program

**Fig. 7:**
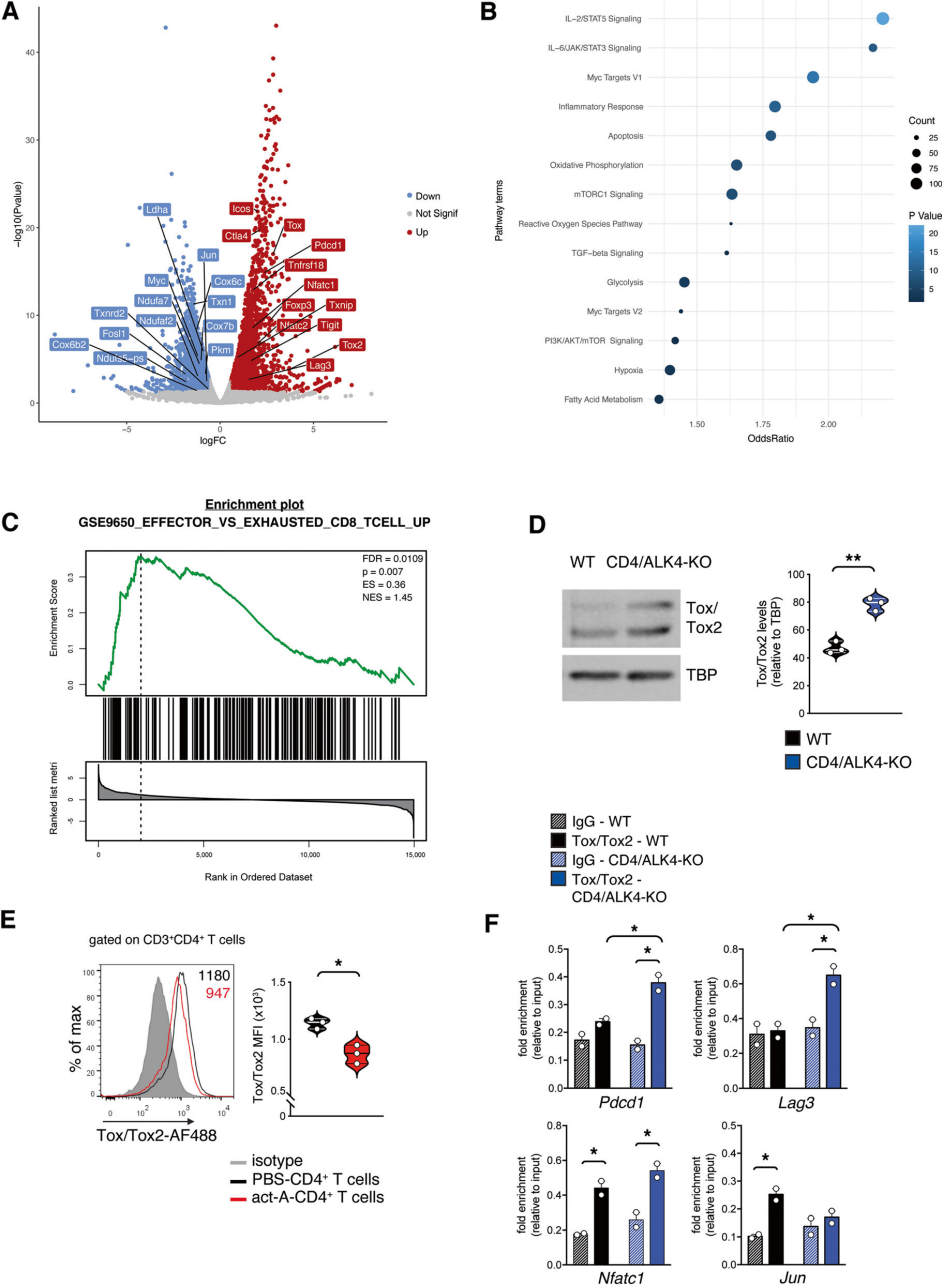
Ablation of activin-A signaling renders CD4^+^ T cells exhausted via a Tox/Tox2-dictated transcriptional program. **A** Changes in gene expression (x-axis) and statistical significance (y-axis) in lung tumor-infiltrating CD4^+^ T cells from CD4/ALK4-KO versus WT mice, presented as a volcano plot. Log2 fold change (x axis) cutoff was set at ±0.58 and *P* value (y axis) cutoff was set at < 0.05. Down: Down-regulated, Up: Up-regulated, Not Signif: Not significant. **B** Pathway analysis of Differentially Expressed Genes. Terms and scores were retrieved from Molecular Signatures Database (MSigDB). **C** GSEA results for Differentially Expressed Genes (DEGs) against a gene set related to CD8^+^ T cell exhaustion, retrieved from Molecular Signatures Database (MSigDB). FDR: False Discovery Rate, p: *p*-value, ES: Enrichment Score, NES: Normalized Enrichment Score. **D** Representative immunoblots showing Tox/Tox2 expression. Quantification of relative Tox/Tox2 protein levels is depicted; TATA binding protein (TBP). Data are mean ± SEM and are representative of 3 independent experiments. **E** Representative flow cytometry plots (left) and cumulative MFI values (right) of Tox/Tox2 expression by ex vivo activin-A- or PBS-treated CD4^+^ T cells from lung tumors of LLC-OVA-inoculated C57BL/6 mice cells. **F** ChIP analyses demonstrating the binding of Tox/Tox2 on the *Pdcd1*, *Lag3*, *Nfatc1* and *Jun* loci. Data are mean ± SEM and are representative of 2–3 independent experiments. Statistical significance was obtained by unpaired Student’s t-test; *P < 0.05 and **P < 0.01

In an effort to define the transcriptional adaptations that account for the establishment of an exhausted phenotype in CD4^+^ Τ cells unresponsive to activin-A signaling, we performed RNA-Seq analysis in lung tumor-infiltrating CD4^+^ Τ cells from CD4/ALK4-KO and WT mice (Fig. 7A). The transcriptomic profile of CD4/ALK4-KO and WT CD4^+^ T cells were distinctly separated by distance clustering (Supplemental Fig. 6A). In agreement with our flow cytometry results, we identified a significant upregulation in several genes encoding for immune checkpoint inhibitory molecules *(*e.g. *Pdcd1, Lag3, Ctla4, Icos, Tigit, Tnfrsf18)* in tumor-infiltrating CD4/ALK4-KO CD4^+^ T cells, compared to WT CD4^+^ T cells (Fig. 7A). On the other hand, the list of downregulated genes was characterized by several factors involved in a plethora of metabolic programs (Fig. 7A). Gene Set Enrichment Analysis (GSEA) revealed that glycolysis, fatty acid and glutathione metabolism and especially oxidative phosphorylation were among the top pathways enriched in genes demonstrating a downregulated expression profile in CD4/ALK4-KO CD4^+^ T cells (Fig. 7B, Supplemental Fig. 6B). Since T cell effector function is considered to be metabolically demanding, we hypothesize that CD4^+^ T cells from CD4/ALK4-KO mice demonstrate either low bioenergetic needs or dysfunctional metabolic mechanisms, probably due to an enhanced exhausted state [41–43]. GSEA, using the Molecular Signatures Database (MSigDB) Hallmark gene set collection [32], revealed a positive correlation with a T cell exhausted signature [44] in CD4/ALK4-KO CD4^+^ T cells, as compared to WT CD4^+^ T cells (Fig. 7C). Associated with a dysfunctional T cell state, these cells exhibited reduced phospho-mTOR protein levels and several downregulated mTOR-related genes, pointing to an overall inactivated mTOR pathway (Supplemental Fig. 6C-D). Consequently, the metabolic regulator Myc (encoded by *Myc*) which is controlled by the m-TOR pathway, as well as, several Myc target-genes, were found downregulated in CD4/ALK4-KO CD4^+^ T cells, as compared to WT CD4^+^ T cells (Fig. 7A, Supplemental Fig. 6C).

Notably, among our top upregulated genes in CD4^+^ T cells from CD4/ALK4-KO mice, were *Tox* and *Tox2* (7.1- and 12.9-folds change, respectively), encoding for the nuclear DNA-binding factor TOX (thymocyte selection-associated HMG-box protein) and TOX2, respectively (Fig. 7A). Several elegant studies have revealed that Tox, and to a lesser extent Tox2, impose T cell exhaustion in the context of chronic infections and cancer [36, 37, 45, 46]. In agreement with the RNA-Seq data, we observed heightened protein expression of Tox and Tox2 in CD4^+^ T cells infiltrating the lung tumors of CD4/ALK4-KO mice, in comparison to WT mice (Fig. 7D). In a reverse approach, ex vivo administration of activin-A reduced the expression of Tox and Tox2 in lung tumor-infiltrating CD4^+^ T cells (Fig. 7E).

Tox directly regulates the expression of *Pdcd1* by binding on its -23 kb enhancer sequence in exhausted CD8^+^ T cells [36, 45]. In an attempt to explore whether Tox and Tox2 participate in the transcriptional regulation of highly upregulated inhibitory receptors in lung tumor-infiltrating CD4^+^ T cells, we performed ChIP analysis. Importantly, we observed that in lung tumor-infiltrating CD4^+^ T cells from CD4/ALK4-KO mice, Tox and Tox2 demonstrated significantly enriched binding on the -23 kb enhancer sequence of *Pdcd1*, compared to their WT counterparts (Fig. 7F). By utilizing published ChIP-Seq data [37] we detected a potential Tox binding region near the transcriptional start site of *Lag3*. Notably, we observed significantly enriched binding of Tox and Tox2 in this regulatory region of *Lag3*, in lung tumor-infiltrating CD4^+^ T cells from CD4/ALK4-KO compared to WT mice (Fig. 7F). Prolonged activation of the NFAT transcription factors is associated with T cell exhaustion, especially in the absence of the canonical AP-1 transcription factor, comprised of c-Fos and c-Jun. [36, 47, 48] In agreement with this notion, our RNA-Seq analysis revealed that several members of the NFAT family were upregulated whereas *Jun* and *Fosl1* were among the significantly downregulated genes in CD4^+^ T cells infiltrating the lung tumors of CD4/ALK4-KO mice, compared to WT mice (Fig. 7A). Using the published ChIP-Seq data [37] we also detected potential Tox binding sites on regulatory regions of *Nfatc1* and *Jun.* Interestingly, Tox and Tox2 demonstrated enriched binding on the *Nfatc1* locus in CD4^+^ T cells from both CD4/ALK4-KO and WT mice (Fig. 7F). On the contrary, we discovered that Tox and Tox2 exhibited significantly enriched binding on the *Jun* locus only in the case of lung tumor-infiltrating CD4^+^ T cells of WT mice (Fig. 7F).

By performing RNA-Seq and ChIP-Seq analyses in CD8^+^ T cells from Tox-KO versus WT mice, Page and colleagues identified 81 genes which were directly regulated by Tox [37]. Notably, the expression of these 81 Tox-dependent genes exhibited an inverse correlation with our RNA-Seq dataset (Supplemental Fig. 6E). This observation further supports our finding that Tox and Tox2 dictate the transcriptional exhaustion program in CD4/ALK4-KO CD4^+^ T cells that infiltrate lung tumors. In summary, our data demonstrate that in the absence of activin-A signaling, CD4^+^ T cells infiltrating lung tumors expressed enhanced levels of Tox and Tox2, which are, at least partly, responsible for the establishment of an exhausted T cell profile.

## Discussion

Although, the majority of cancer immunotherapies center around exploiting the cytotoxic properties of CD8^+^ T cells, the potential role of CD4^+^ T helper cells remains ill-defined. Hence, the identification of factors that can direct persistent, effective CD4^+^ T cell-mediated anti-tumor responses may set the groundwork for future efficacious cancer immunotherapies. In this study, we explored the effects of the pleiotropic cytokine activin-A, on shaping anti-tumor CD4^+^ T cell-mediated responses in the context of lung cancer.

In lung cancer, activin-A exhibits both pro- and anti-tumorigenic properties, underlying a cell type-specific and cancer stage-related effect [18, 20, 49]. Ablation of activin-A’s expression in lung cancer cells, led to reduced formation of lung metastasis along with prolonged survival of tumor-bearing hosts [20]. In agreement to the aforementioned findings, inactivation of activin-A signaling in lung cancer cells increased the therapeutic efficacy of platinum-based chemotherapy in tumor-bearing mice [18]. On the other hand, Shurin et al. demonstrated that adoptive transfer of activin-A-treated DCs in both melanoma and lung carcinoma models inhibited tumor growth and enhanced the overall survival of tumor-bearing mice, partly through the generation of tumor-specific IFN-γ-producing T cells [49].

Our group, has extensively studied the direct effect of activin-A on CD4^+^ T cells during allergic and autoimmune responses [50–52]. In experimental autoimmune encephalomyelitis, activin-A suppressed the pathogenic signature and the encephalitogenic functions of Th17 cells, at least partly through the enhanced expression of the anti-inflammatory ectonucleotidases CD73 and CD39 [50]. Moreover, we discovered that activin-A instructed the conversion of effector Th2 cells towards IL-10-expressing T cells with immunoregulatory properties in the context of asthmatic responses [51, 52]. Nevertheless, there is a significant gap in our knowledge regarding the role of activin-A in the modulation of anti-tumor CD4^+^ T cell-mediated immune responses in the context of lung cancer. In this study, we demonstrated that in vivo administration of activin-A limited the formation of primary lung tumors, as well as, melanoma metastases in the lungs, effects associated with a marked enhancement of anti-tumor effector T cell responses. The anti-tumorigenic properties of activin-A were mediated by the increased infiltration of effector CD4^+^ T cells in lung tumors, concomitantly with the diminished presence of Treg cells in the tumor microenvironment. The ratio of effector to regulatory T cells infiltrating tumors correlates with better prognosis, response to treatment and overall survival of patients with several cancer types, including NSCLC [9]. However, tumor-infiltrating T cells are rendered dysfunctional or exhausted due to the establishment of a strong immunosuppressive microenvironment [53]. Importantly, exogenous administration of activin-A reversed part of this dysfunctional T cell phenotype and granted CD4^+^ T cells with strong effector properties. Furthermore, ex vivo treatment of tumor-infiltrating CD4^+^ T cells with activin-A bestowed them with robust effector properties which were maintained upon therapeutic transfer in vivo and empowered them to protect against lung cancer progression.

Intriguingly, activin-A is detected in significant levels in the serum and tumor microenvironment of lung cancer patients [17–20]. In line with the aforementioned findings, in our lung tumor setting activin-A was elevated in the serum and lungs of tumor-bearing mice, perhaps as part of a defense mechanism, that aims to target CD4^+^ T cells and endow them with enhanced effector properties. For this, we examined the phenotype and function of tumor-infiltrating CD4^+^ T cells which cannot transduce activin-A signals through ALK4, during lung tumor development in vivo. We observed that these cells exhibited higher levels of exhaustion, exemplified by reduced expression of T effector cytokines, along with enhanced expression of immunosuppressive factors and were unable to instruct tumor-infiltrating CD8^+^ T cells to exert competent cytotoxic functions. Notably, our findings were validated in a translational setting, utilizing primary human NSCLC lung tumor-infiltrating CD4^+^ T cells. Ex vivo administration of activin-A on human lung tumor-infiltrating CD4^+^ T cells partially reversed their functional impairment, evidenced by the enhanced immunostimulatory potential towards autologous CD4^+^ and CD8^+^ T cells thus resembling the anti-tumorigenic effects observed in murine models.

Dysfunctional T cells display a defective metabolic profile attributed to both cell intrinsic metabolic alterations, as well as, the unfavourable metabolic milieu in the tumor microenvironment [54]. RNA-Seq analysis revealed that disruption of activin-A signaling on lung tumor-infiltrating CD4^+^ T cells was associated with a reduction in gene sets related to the metabolic function of these cells. Our attempts to define the molecular alterations that are imposed by the absence of activin-A signaling on lung tumor-infiltrating CD4^+^ T cells, unraveled the transcription factors Tox and Tox2 partly responsible for the establishment of a molecular program of exhaustion [44]. Indeed, we found that Tox and Tox2 directly control the transcriptional regulation of *Pdcd1* and *Lag3* expression, largely determining the functional incompetence of tumor-infiltrating CD4^+^ T cells from CD4/ALK4-KO mice. Tox and Tox2, are extensively studied in CD8^+^ T cells, in the context of chronic infections and cancer [36, 45, 55, 56]. Interestingly, single-cell RNA-Seq data analysis of T cells from NSCLC patients revealed that Tox is highly enriched in exhausted CD4^+^ and CD8^+^ T cell populations, underlying its correlation with a hypo-responsive gene signature [57]. In agreement with our findings, Tox and Tox2 were upregulated in exhausted PD-1^high^TIM3^high^ tumor-infiltrating CD8^+^ T cells expressing chimeric antigen receptors (CAR). Additionally, CAR T cells deficient in both Tox and Tox2 were more effective than WT in augmenting effector cytokine expression, reducing inhibitory receptor expression, controlling tumor growth and extending survival of tumor-bearing mice [36]. In support of our findings, enforced Tox expression induced an exhaustion transcriptional signature in CD8^+^ T cells during viral infection and skewed T cells away from effector and memory phenotypes [45].

Notably, transcriptional and epigenetic alterations in Tox deficient T cells were negatively associated with transcription factor networks downstream of the TCR signaling, among which was NFAT2, also shown to directly regulate Tox expression [45]. Interestingly, we discovered that in tumor-infiltrating CD4^+^ T cells Tox and Tox2 bind on the genetic locus of NFAT2, suggesting the existence of a positive feedback loop in the regulation of gene expression between NFAT2 and Tox. On the contrary, Tox and Tox2 did not bind on the genetic locus of Jun in tumor-infiltrating CD4^+^ T cells from CD4/ALK4-KO mice. Given that the family of NFAT transcription factors are known to promote T cell exhaustion, especially in the absence of the canonical AP-1 family transcription factor [36, 47, 48], we believe that the expression of Jun was negatively regulated in order to facilitate the establishment of T cell exhaustion in lung cancer. Since CD4^+^ T cell exhaustion is far less studied, especially in the context of cancer, prospective research is required to reveal the role of these transcription factors in their full extent. Additionally, although this is one of the first attempts to unveil the role of activin-A in exhausted CD4^+^ T cells, there is still a significant gap in our knowledge regarding the precise signaling pathways which participate in and mediate the anti-tumorigenic properties of activin-A. Since, canonical (SMAD2/3-mediated) and non-canonical activin-A signaling pathways [16] could be utilized by multiple factors other than activin-A, we anticipate their participation in different and even opposing signaling events in exhausted CD4^+^ T cells and it is our intention to explore them in future studies. Overall, our study has laid the foundation for addressing key questions in the field of lung cancer immunology and foreseeing the exploitation of activin-A-conditioned CD4^+^ T cells in future therapeutic modalities, in the context of adoptive T cell therapy.

## Conclusions

In summary, our studies reveal that the cytokine activin-A enhances anti-tumor immunity orchestrated by CD4^+^ T cells, in the context of lung cancer. Additionally, CD4^+^ T cell-specific ablation of activin-A signaling during lung tumor development, establishes an exhaustion molecular signature on CD4^+^ T cells, dictated by the transcription factors Tox and Tox2. Furthermore, we uncovered the potential of activin-A to strengthen T cell responses of human NSCLC patient tumor-infiltrating cells. Here, we introduce activin-A as a novel immunomodulatory factor which can reinvigorate exhausted, lung tumor-infiltrating CD4^+^ T cells. Thus, the impact of this study lies on the envision of utilizing activin-A-conditioned CD4^+^ T cells in future therapeutic modalities of lung cancer.

## Supplementary Information

**Additional file 1: Supplemental Fig. 1.** In vivo administration of activin-A attenuates the development and progression of melanoma lung metastases. **Supplemental Fig. 2.** Activin-A represses the expression of inhibitory receptors on melanoma lung metastases-infiltrating CD4^+^ cells. **Supplemental Fig. 3.** Disruption of activin-A’s signaling on CD4^+^ T cells accelerates tumor progression in a chemically-induced model of lung cancer. **Supplemental Fig. 4.** Activin-A alters the expression of inhibitory receptors on lung tumor-infiltrating CD4^+^ T cells. **Supplemental Fig. 5.** Activin-A unveils different effects on T cells infiltrating lung tumors compared to adjacent healthy lung tissue. **Supplemental Fig. 6.** Ablation of activin-A signaling imposes metabolic dysfunction on lung tumor-infiltrating CD4^+^ T cells. **Supplemental Table 1.** NSCLC patient characteristics.

## Data Availability

The RNA-Seq datasets generated in the current study have been deposited in the GEO Dataset repository (accession no.GSE183544).

## Abbreviations

CAR: Chimeric antigen receptor
ChIP: Chromatin immunoprecipitation
ChIP-Seq: ChIP-Sequencing
CM: Condition media
GSEA: Gene Set Enrichment Analysis
H&E: Hematoxylin & eosin
i.p.: Intraperitoneally
i.v.: Intravenously
LLC: Lewis Lung Carcinoma
MSigDB: Molecular Signatures Database
NSCLC: Non-small cell lung carcinoma
OVA: Ovalbumin
PBMCs: Peripheral blood mononuclear cells
RNA-Seq: RNA-Sequencing
TBP: TATA-binding protein
TILs: Tumor-infiltrating lymphocytes
TOX: Thymocyte selection-associated HMG-box protein
WT: Wild-type
