# Supplementary material for: Activin-A impedes the establishment of CD4+ T cell exhaustion and enhances anti-tumor immunity in the lung
Source: J Exp Clin Cancer Res. 2021 Sep 21;40:295. doi: 10.1186/s13046-021-02092-5 (PMC8454162; doi:10.1186/s13046-021-02092-5)
Supplement: Supplementary file 1 — Additional file 1: Supplemental Fig. 1. In vivo administration of activin-A attenuates the development and progression of melanoma lung metastases. Supplemental Fig. 2. Activin-A represses the expression of inhibitory receptors on melanoma lung metastases-infiltrating CD4+ cells. Supplemental Fig. 3. Disruption of activin-A’s signaling on CD4+ T cells accelerates tumor progression in a chemically-induced model of lung cancer. Supplemental Fig. 4. Activin-A alters the expression of inhibitory receptors on lung tumor-infiltrating CD4+ T cells. Supplemental Fig. 5. Activin-A unveils different effects on T cells infiltrating lung tumors compared to adjacent healthy lung tissue. Supplemental Fig. 6. Ablation of activin-A signaling imposes metabolic dysfunction on lung tumor-infiltrating CD4+ T cells. Supplemental Table 1. NSCLC patient characteristics. [file 13046_2021_2092_MOESM1_ESM.pdf]

Supplementary material for:

Activin-A impedes the establishment of CD4$^+$ T cell exhaustion and enhances anti-tumor immunity in the lung

Ioannis Morianos[1], Aikaterini Tsitsopoulou[1], Konstantinos Potaris[2], Dimitrios Valakos[3], Ourania Fari[1†], Giannis Vatsellas[4], Clementine Bostantzoglou[5], Andreas Photiades[6], Mina Gaga[6], Georgina Xanthou[1] and Maria Semitekolou[1*]

[1]Cellular Immunology Laboratory, Biomedical Research Foundation of the Academy of Athens (BRFAA), Athens, 11527, Greece; [2]Department of Thoracic Surgery, Athens Chest Hospital 'Sotiria', Athens, 11527, Greece; [3]Molecular Biology Laboratory, BRFAA, Athens, 11527, Greece; [4]Greek Genome Center, BRFAA, Athens, 11527, Greece; [5]Intensive Care Unit, Athens Chest Hospital 'Sotiria', Athens, 11527, Greece; [6]7th Respiratory Medicine Department and Asthma Center, Athens Chest Hospital 'Sotiria', Athens, 11527, Greece.

[†]Current address: Institute of Cancer Research, Department of Medicine I, Comprehensive Cancer Center, Medical University of Vienna, Vienna, 1090, Austria.

*Corresponding author:

Maria Semitekolou, PhD

Cellular Immunology Laboratory,

Biomedical Research Foundation of the Academy of Athens,

Athens 115 27 Greece

Tel: +30-210-6597 336

Email: msemi@bioacademy.gr

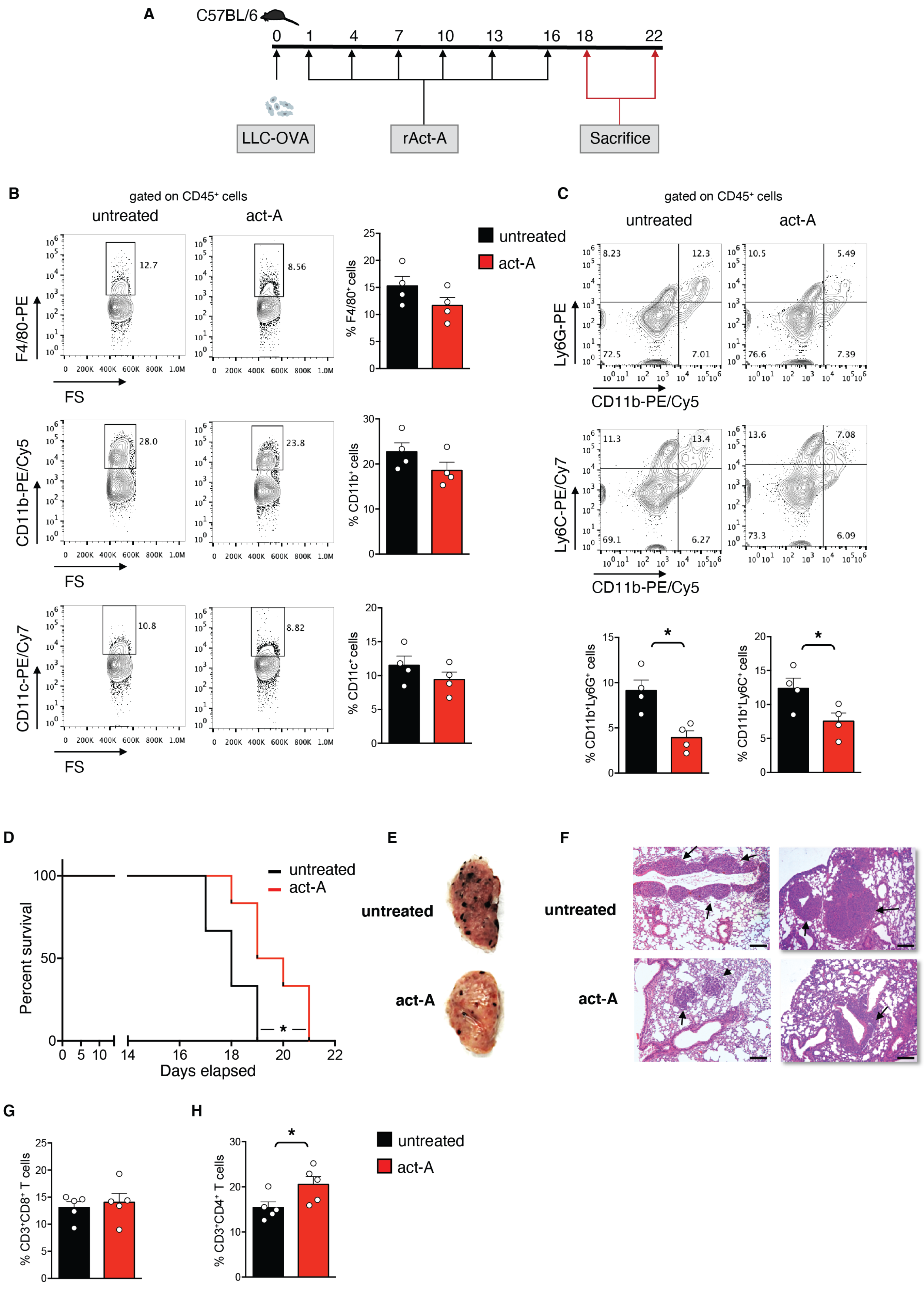

A
C57BL/6
0 1 4 7 10 13 16 18 22
LLC-OVA
rAct-A
Sacrifice
B
gated on CD45⁺ cells
untreated
act-A
F4/80-PE
CD11b-PE/Cy5
CD11c-PE/Cy7
FS
12.7
8.56
28.0
23.8
10.8
8.82
% F4/80⁺cells
% CD11b⁺cells
% CD11c⁺cells
untreated
act-A
C
gated on CD45⁺ cells
untreated
act-A
Ly6G-PE
Ly6C-PE/Cy7
CD11b-PE/Cy5
8.23
12.3
72.5
7.01
10.5
5.49
76.6
7.39
11.3
13.4
69.1
6.27
13.6
7.08
73.3
6.09
% CD11b⁺Ly6G⁺ cells
% CD11b⁺Ly6C⁺ cells
D
Percent survival
Days elapsed
untreated
act-A
E
untreated
act-A
F
untreated
act-A
G
% CD3⁺CD8⁺ T cells
H
% CD3⁺CD4⁺ T cells
untreated
act-A

Supplemental figure 1. *In vivo* administration of activin-A attenuates the development and progression of melanoma lung metastases.

(**A**) Experimental protocol utilized. (**B**) Representative flow cytometry plots (left) and cumulative percentages of F4/80$^+$ macrophages, CD11b$^+$ myeloid and CD11c$^+$ dendritic cells among CD45$^+$ lung tumor infiltrating cells (right). (**C**) Representative flow cytometry plots (left) and cumulative percentages of CD11b$^+$Ly6G$^+$ granulocytic and CD11b$^+$Ly6C$^+$ monocytic myeloid derived suppressor cells among CD45$^+$ lung tumor infiltrating cells (right). (**D**) Survival plot of activin-A-treated or untreated B16F10-tumor bearing mice (*n*=6). (**E**) Representative macroscopic images of B16F10-tumor bearing lung lobes. (**F** Representative photomicrographs of H&E-stained lung sections (scale bars: 100μm). (**G**) Cumulative percentages of CD3$^+$CD8$^+$ T cells among CD45$^+$ lung tumor infiltrating cells. (**H**) Cumulative percentages of CD3$^+$CD4$^+$ T cells among CD45$^+$ lung tumor infiltrating cells. Data are representative of 4-5 independent experiments. Statistical significance was obtained by Log-rank (Mantel-Cox) test and unpaired Student's t-test; *$P < 0.05$.

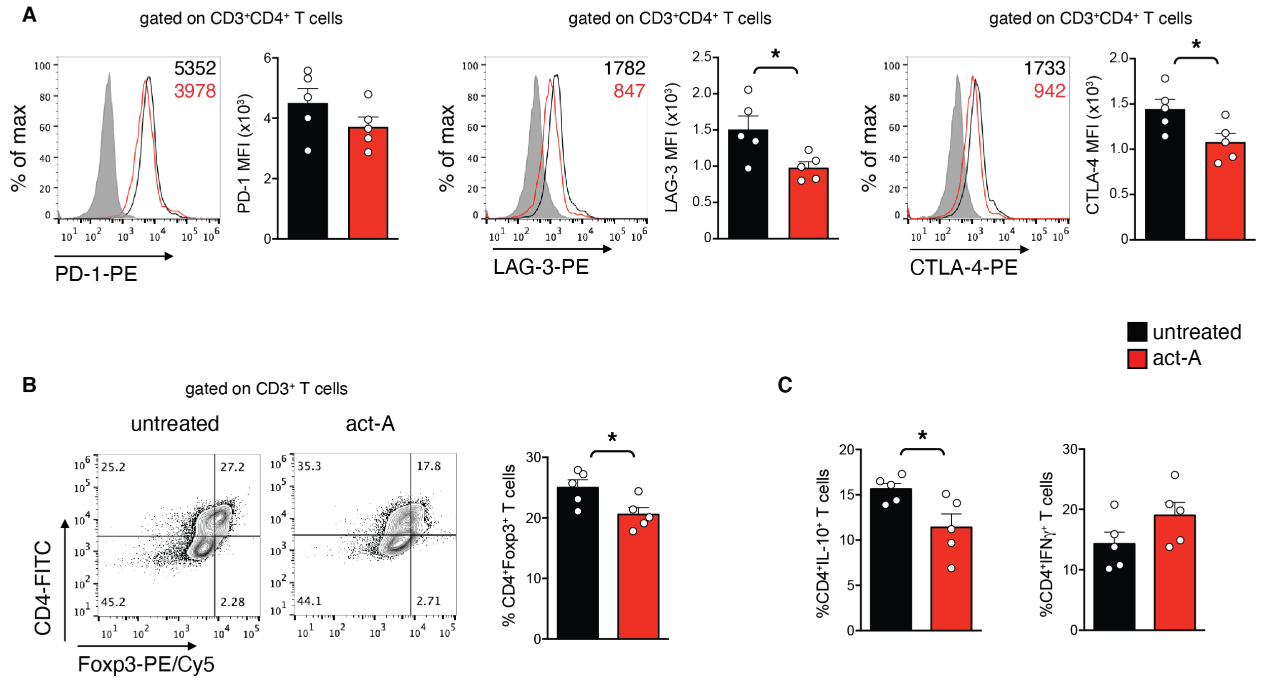

Supplemental figure 2. **Activin-A represses the expression of inhibitory receptors on melanoma lung metastases-infiltrating CD4$^+$ T cells.**

(**A**) Representative flow cytometry plots (left) and cumulative MFI values of PD-1, LAG-3 or CTLA-4 expression by CD3$^+$CD4$^+$ T cells infiltrating lung tumors (right) from activin-A-treated or untreated B16F10-tumor bearing mice. Numbers in plots indicate MFI values. Shaded histograms represent isotype controls. (**B**) Representative flow cytometry plots (left) and cumulative percentages of Foxp3-expressing cells among CD3$^+$CD4$^+$ T cells infiltrating lung tumors (right). (**C**) Cumulative percentages of IL-10- or IFN-γ-expressing cells among CD3$^+$CD4$^+$ T cells infiltrating lung tumors. Data are representative of 5 independent experiments. Statistical significance was obtained by unpaired Student's t-test; *$P < 0.05$.

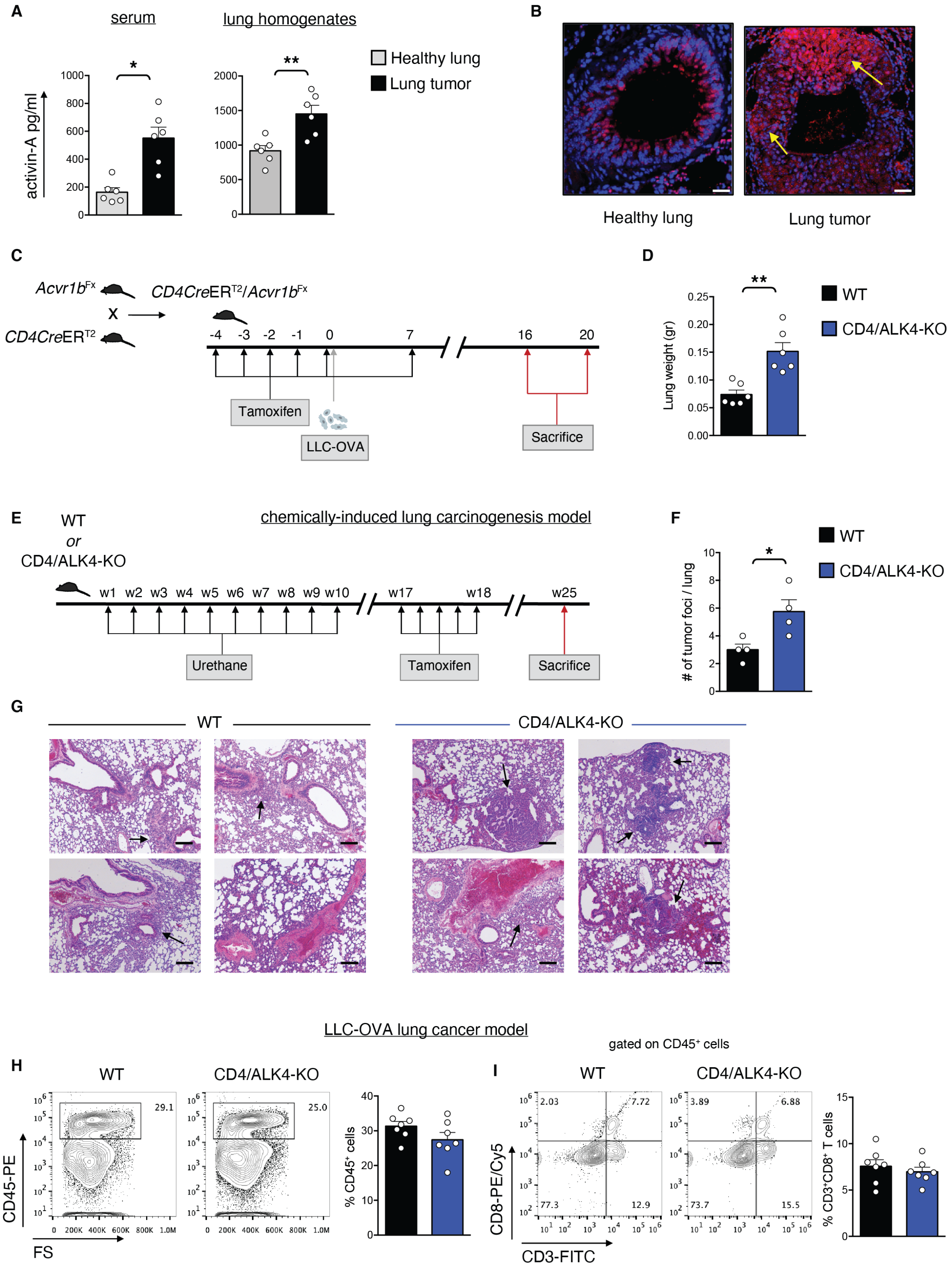
A
serum
lung homogenates
activin-A pg/ml
Healthy lung
Lung tumor
B
Healthy lung
Lung tumor
C
Acvr1b^Fx
X
CD4CreER^T2
CD4CreER^T2/Acvr1b^Fx
-4 -3 -2 -1 0 7 16 20
Tamoxifen
LLC-OVA
Sacrifice
D
Lung weight (gr)
WT
CD4/ALK4-KO
E
WT
or
CD4/ALK4-KO
chemically-induced lung carcinogenesis model
w1 w2 w3 w4 w5 w6 w7 w8 w9 w10 w17 w18 w25
Urethane
Tamoxifen
Sacrifice
F
# of tumor foci / lung
WT
CD4/ALK4-KO
G
WT
CD4/ALK4-KO
LLC-OVA lung cancer model
H
WT
CD4/ALK4-KO
CD45-PE
FS
29.1
25.0
% CD45+ cells
I
gated on CD45+ cells
WT
CD4/ALK4-KO
CD8-PE/Cy5
CD3-FITC
2.03
7.72
77.3
12.9
3.89
6.88
73.7
15.5
% CD3+CD8+ T cells

Supplemental figure 3. **Disruption of activin-A's signaling on CD4^{+} T cells accelerates tumor progression in a chemically-induced model of lung cancer.**

(**A**) Activin-A levels in serum and lung homogenates from healthy and LLC tumor-bearing mice. Data are mean ± SEM of triplicate wells and are representative of 6 independent experiments. (**B**) Representative confocal microscopy images of lung sections from healthy and LLC tumor-bearing mice stained with an antibody against activin-A (red). Nuclei are stained blue with DAPI (scale bars: 20μm). (**C**) Experimental protocol utilized. (**D**) Lung weight measurements of CD4/ALK4-KO and WT LLC-tumor bearing mice (*n*=6). (**E**) Experimental protocol utilized. (**F**) Tumor foci measurements of CD4/ALK4-KO and WT lung-tumor bearing mice (*n*=4). (**G**) Representative photomicrographs of H&E-stained lung sections (scale bars: 100μm). (**H**) Representative flow cytometry plots (left) and cumulative percentages of CD45^{+} lung tumor infiltrating cells (right). Data are representative of 7 independent experiments. (**I**) Representative flow cytometry plots (left) and cumulative percentages of CD3^{+}CD8^{+} T cells among CD45^{+} lung tumor infiltrating cells (right). Data are representative of 7 independent experiments. Statistical significance was obtained by unpaired Student's t-test; *$P < 0.05$ and **$P < 0.01$.

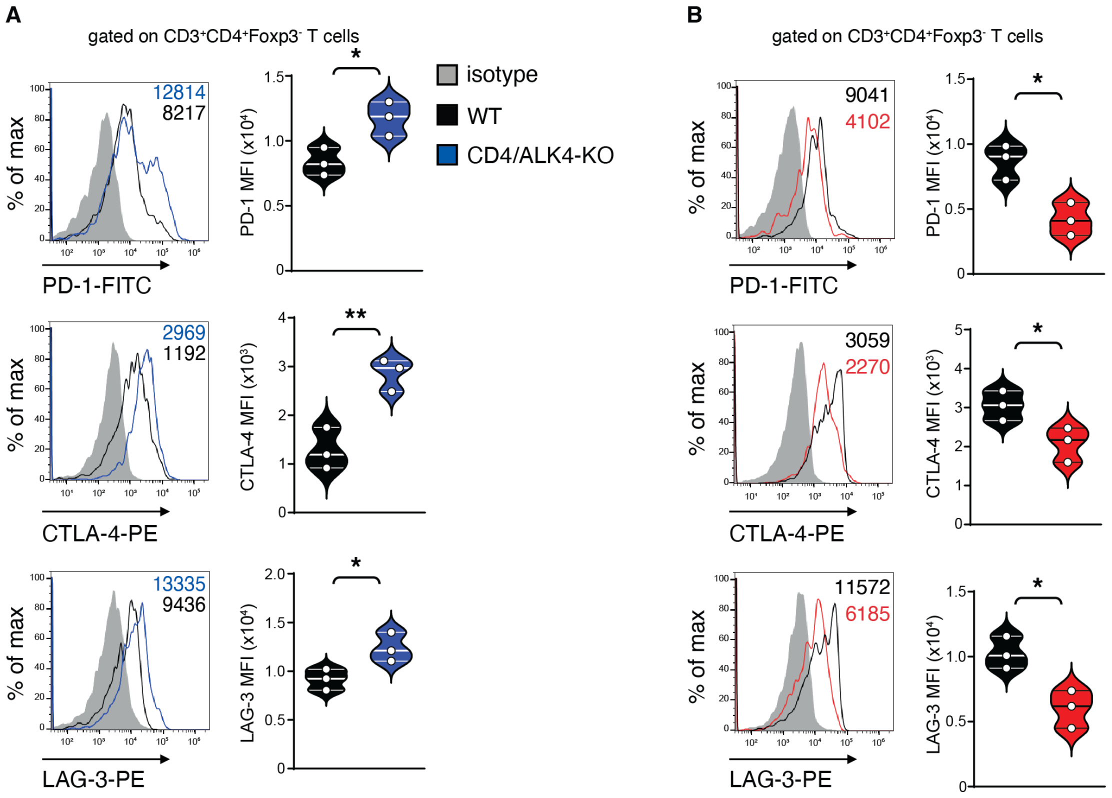

Supplemental figure 4. **Activin-A alters the expression of inhibitory receptors on lung tumor-infiltrating CD4$^+$ T cells.**

(**A**) Representative flow cytometry plots (left) and cumulative MFI values of PD-1, CTLA-4 or LAG-3 expression by CD3$^+$CD4$^+$Foxp3$^-$ T cells infiltrating lung tumors (right) from CD4/ALK4-KO or WT LLC-OVA-tumor bearing mice. (**B**) Representative flow cytometry plots (left) and cumulative MFI values of PD-1, CTLA-4 or LAG-3 expression by CD3$^+$CD4$^+$Foxp3$^-$ T cells infiltrating lung tumors (right) from activin-A-treated or untreated LLC-OVA-tumor bearing mice. Numbers in plots indicate MFI values. Shaded histograms represent isotype controls. Data are representative of 3 independent experiments. Statistical significance was obtained by unpaired Student's t-test; *$P < 0.05$ and **$P < 0.01$.

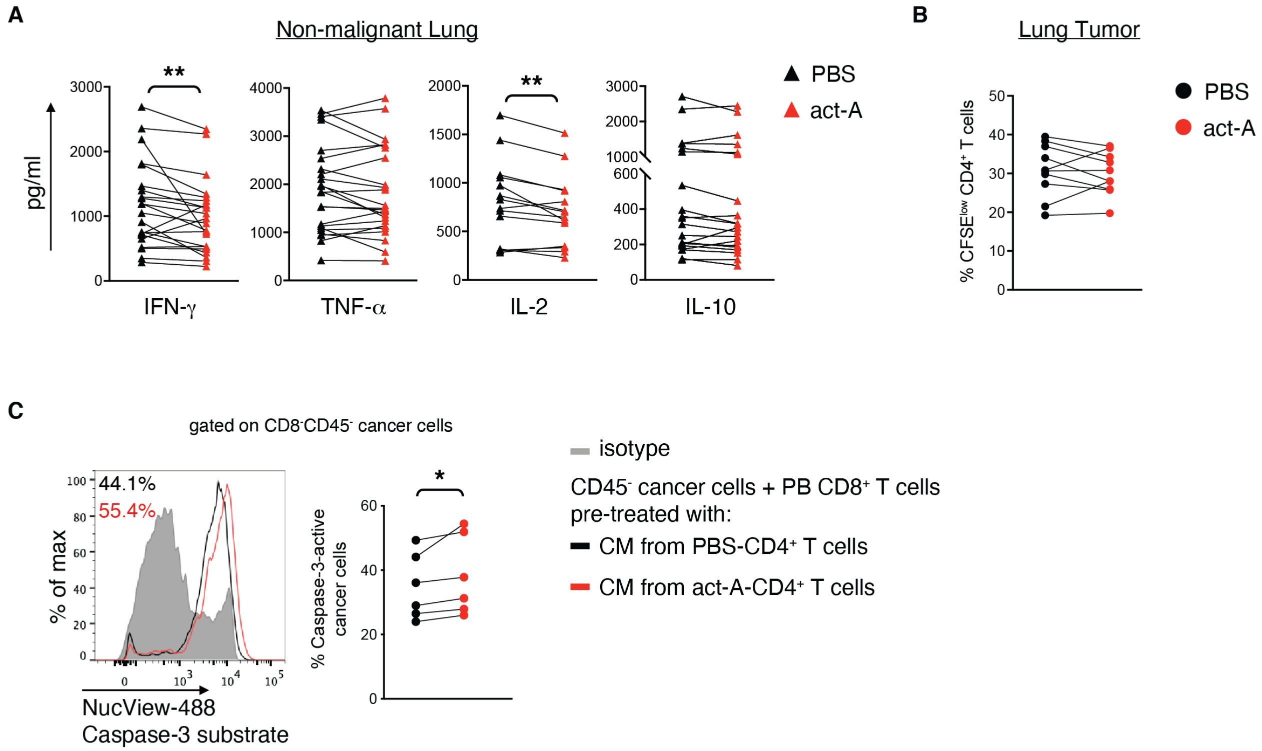

Supplemental figure 5. Activin-A unveils different effects on T cells infiltrating lung tumors compared to adjacent healthy lung tissue.

(**A**) Primary lung infiltrating leukocytes were isolated from, adjacent to tumors, non-malignant tissues form NSCLC patients and stimulated with antibodies against CD3/CD28 in the presence of activin-A or PBS. Cytokine levels in culture supernatants of activin-A- or PBS-treated non-malignant lung infiltrating leukocytes (*n*= 20-26 donors). Data are mean ± SEM of triplicate wells. (**B**) Activin-A- or PBS-treated human lung tumor infiltrating $CD4^+$ T cells were generated as in Fig. 6A and labelled with CellTrace-CFSE. Cumulative data of $CD4^+$ T cell proliferation, measured by flow cytometry are depicted (*n*= 10 donors). (**C**) $CD45^-$ lung cancer cells were co-cultured with autologous PB $CD8^+$ T cells pre-treated with CM from act-A- or PBS-$CD4^+$ T cell cultures. Representative flow cytometry plot (left) and cumulative percentages (right) depicting caspase-3 activity of $CD45^-$ lung cancer cells (*n*=6). Statistical significance was obtained by Wilcoxon matched-pairs signed rank test; *P < 0.05 and **P < 0.01.

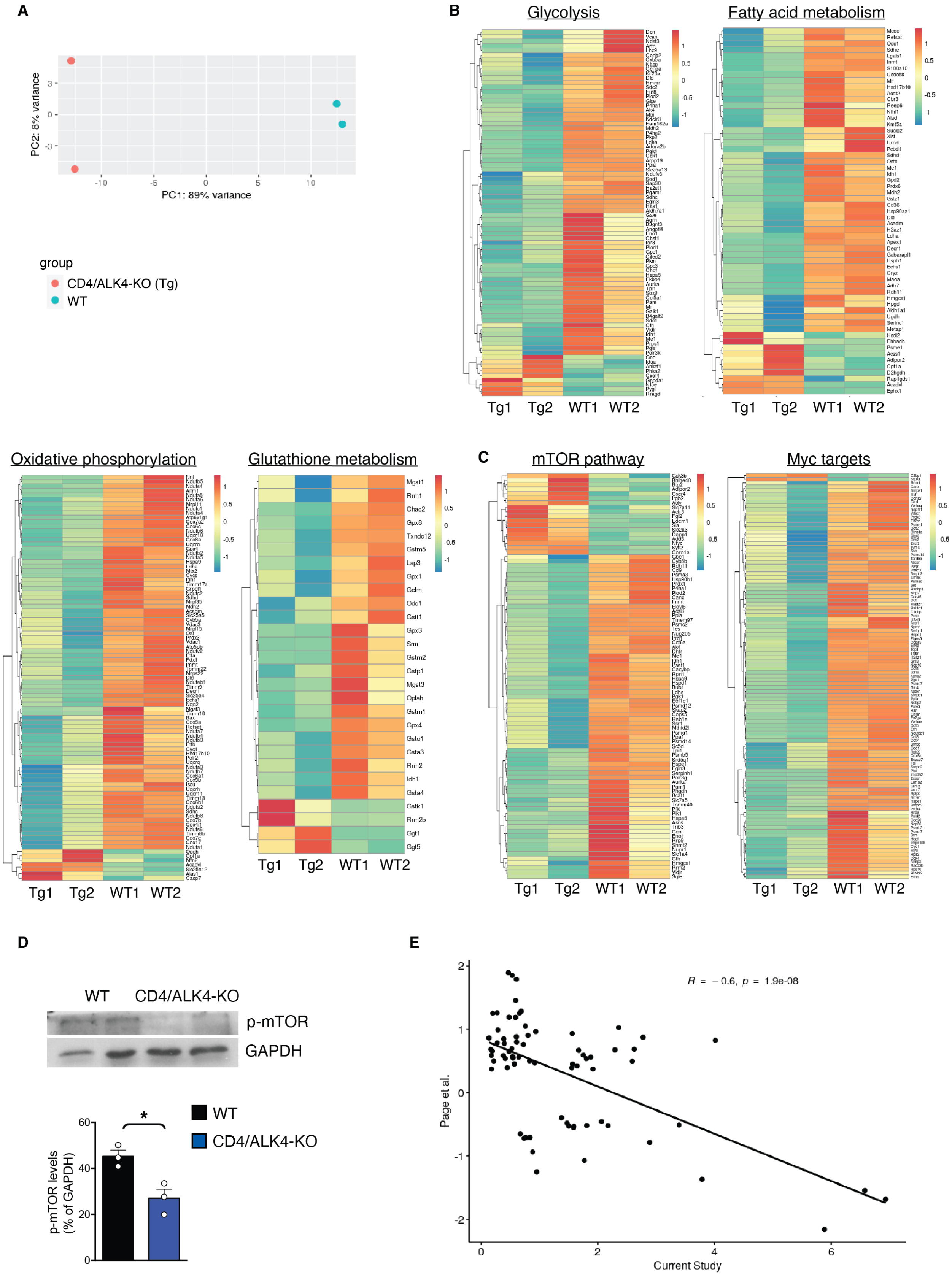
A
PC2: 8% variance
PC1: 89% variance
group
CD4/ALK4-KO (Tg)
WT
B
Glycolysis
Fatty acid metabolism
Tg1 Tg2 WT1 WT2
Oxidative phosphorylation
Glutathione metabolism
C
mTOR pathway
Myc targets
D
WT CD4/ALK4-KO
p-mTOR
GAPDH
p-mTOR levels (% of GAPDH)
WT
CD4/ALK4-KO
E
R = −0.6, p = 1.9e-08
Page et al.
Current Study

Supplemental figure 6. **Ablation of activin-A signaling imposes metabolic dysfunction on lung tumor infiltrating $CD4^+$ T cells.**

(**A**) Principle Component Analysis (PCA) for normalized gene counts in lung tumor infiltrating $CD4^+$ T cells (each dot represents an individual $CD4^+$ T cell sample derived from either CD4/ALK4-KO or WT mice). Counts were normalized to log2 scale. (**B**) Heatmap depicting z-score differences among the analyzed samples regarding glycolysis, fatty-acid metabolism, oxidative phosphorylation, glutathione metabolism genes. (**C**) Heatmap depicting z-score differences among the analyzed samples regarding mTOR and Myc signaling pathways genes. (**D**) Representative immunoblots showing p-mTOR expression. Quantification of relative p-mTOR protein levels is depicted. Data are mean ± SEM and are representative of 3 independent experiments. (**E**) Correlation analysis regarding fold changes of Tox-regulated genes according to Page *et al.*, obtained from the respective publication and our RNA-Seq analysis. Applied is Pearson's correlation coefficient analysis. Statistical significance was obtained by unpaired Student's t-test; $^*P < 0.05$.

Supplemental table 1. NSCLC patient characteristics (*n*=38)

**Age**	Mean	70.2
	Range	51-86
**Gender (%)**	Female	11 (28.9)
	Male	27 (71.1)
**Histology (%)**	Adenocarcinoma	21 (55.3)
	Squamous cell carcinoma	16 (42.1)
	Other*	1 (2.6)
**Smoking status (%)**	Current	27 (71.1)
	Former	5 (13.1)
	Never	6 (15.8)
**TNM Stage (%)**	IA (T1a/b/cN0M0)	13 (34.2)
	IB (T2aNOM0)	12 (31.6)
	IIA (T2bN0M0)	3 (7.9)
	IIB (cT3N0M0, cT1N1M0)	5 (13.15)
	IIIA (T2bN2M0, T4N1M0, T4N0M0, T3cN1M0)	5 (13.15)
**Concomitant Disease (%)**	COPD**	11 (28.9)
	Heart Disease	16 (42.1)
	Diabetes	6 (15.8)
	None	12 (31.6)

*Other refers to Neuroendocrine tumors
**Chronic Obstructive Pulmonary Disease
